# Cost-effectiveness of lipid lowering with statins and ezetimibe in chronic kidney disease

**DOI:** 10.1016/j.kint.2019.01.028

**Published:** 2019-07

**Authors:** Iryna Schlackow, Seamus Kent, William Herrington, Jonathan Emberson, Richard Haynes, Christina Reith, Rory Collins, Martin J. Landray, Alastair Gray, Colin Baigent, Borislava Mihaylova, SHARP Collaborative Group, R. Collins, C. Baigent, M.J. Landray, C. Bray, Y. Chen, A. Baxter, A. Young, M. Hill, C. Knott, A. Cass, B. Feldt-Rasmussen, B. Fellström, D.E. Grobbee, C. Grönhagen-Riska, M. Haas, H. Holdaas, L.S. Hooi, L. Jiang, B. Kasiske, U. Krairittichai, A. Levin, Z.A. Massy, V. Tesar, R. Walker, C. Wanner, D.C. Wheeler, A. Wiecek, T. Dasgupta, W. Herrington, D. Lewis, M. Mafham, W. Majoni, C. Reith, J. Emberson, S. Parish, D. Simpson, J. Strony, T. Musliner, L. Agodoa, J. Armitage, Z. Chen, J. Craig, D. de Zeeuw, J.M. Gaziano, R. Grimm, V. Krane, B. Neal, V. Ophascharoensuk, T. Pedersen, P. Sleight, J. Tobert, C. Tomson

**Affiliations:** 1 Health Economics Research Centre, Nuffield Department of Population Health, University of Oxford, UK; 2 Clinical Trial Service Unit and Epidemiological Studies Unit, Nuffield Department of Population Health, University of Oxford, UK; 3 Medical Research Council Population Health Research Unit, Nuffield Department of Population Health, University of Oxford, UK; 4 Centre for Primary Care and Public Health, Blizard Institute, Barts and The London School of Medicine and Dentistry, Queen Mary University of London, UK

**Keywords:** chronic kidney disease, cost-effectiveness, ezetimibe, health care costs, quality-adjusted life years, statin

## Abstract

Statin-based treatments reduce cardiovascular disease (CVD) risk in patients with non-dialysis chronic kidney disease (CKD), but it is unclear which regimen is the most cost-effective. We used the Study of Heart and Renal Protection (SHARP) CKD-CVD policy model to evaluate the effect of statins and ezetimibe on quality-adjusted life years (QALYs) and health care costs in the United States (US) and the United Kingdom (UK). Net costs below $100,000/QALY (US) or £20,000/QALY (UK) were considered cost-effective. We investigated statin regimens with or without ezetimibe 10 mg. Treatment effects on cardiovascular risk were estimated per 1-mmol/L reduction in low-density lipoprotein (LDL) cholesterol as reported in the Cholesterol Treatment Trialists’ Collaboration meta-analysis, and reductions in LDL cholesterol were estimated for each statin/ezetimibe regimen. In the US, atorvastatin 40 mg ($0.103/day as of January 2019) increased life expectancy by 0.23 to 0.31 QALYs in non-dialysis patients with stages 3B to 5 CKD, at a net cost of $20,300 to $78,200/QALY. Adding ezetimibe 10 mg ($0.203/day) increased life expectancy by an additional 0.05 to 0.07 QALYs, at a net cost of $43,600 to $91,500/QALY. The cost-effectiveness findings and policy implications in the UK were similar. In summary, in patients with non-dialysis-dependent CKD, the evidence suggests that statin/ezetimibe combination therapy is a cost-effective treatment to reduce the risk of CVD.

## Graphical abstract

**Figure:**
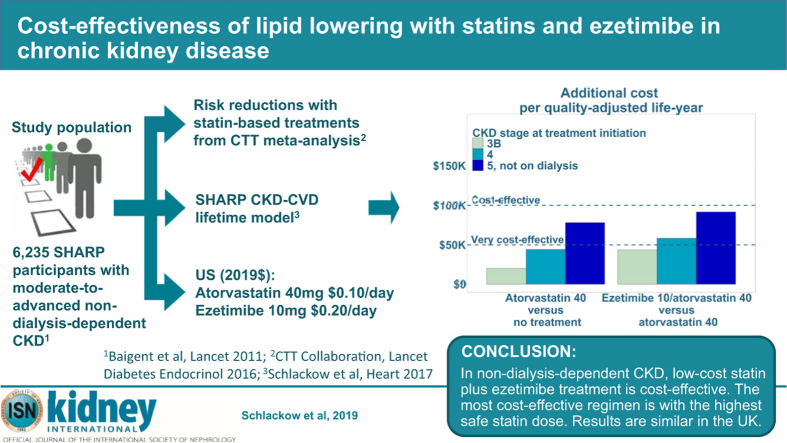

see commentary on page 22

With over 250 million people affected worldwide, CKD is a common disease,^1^, ^2^ and its prevalence is expected to increase further with rising levels of obesity and diabetes and an aging population. In the US, approximately 7% of adults have stage 3-5 CKD.^3^ People with reduced kidney function have increased cardiovascular risk,^4^, ^5^ which is a key treatment target.

The SHARP trial showed that lowering LDL cholesterol with a combination of simvastatin 20 mg plus ezetimibe 10 mg daily for about 5 years safely reduced the risk of major atherosclerotic events (i.e., nonfatal myocardial infarction or coronary death, nonhemorrhagic stroke, or arterial revascularization procedure) in nondialysis patients with moderate-to-advanced CKD.^6^ Subsequently, a large meta-analysis of individual participant data from 28 trials of statin therapy, or, in the case of SHARP, statin plus ezetimibe, was coordinated by the Cholesterol Treatment Trialists’ (CTT) Collaboration.^7^ This showed that the relative reduction in major vascular events (i.e., major atherosclerotic event, noncoronary cardiac death, or hemorrhagic stroke) per 1 mmol/L reduction in LDL cholesterol ranged from 22% among participants with estimated glomerular filtration rate (eGFR) of ≥60 ml/min per 1.73 m^2^ to 15% among nondialysis participants with eGFR <30 ml/min per 1.73 m^2^, with a trend toward smaller risk reductions with lower eGFR and no evidence of clinical efficacy in dialysis patients. By design, SHARP did not assess the separate clinical effects of ezetimibe, but evidence of the effects on vascular outcomes of adding ezetimibe to simvastatin, albeit in patients with an acute coronary syndrome and without established CKD, has been provided by the Improved Reduction of Outcomes: Vytorin Efficacy International Trial (IMPROVE-IT).^8^ In this trial, for each mmol/L reduction in LDL cholesterol, the relative reduction in vascular events resulting from ezetimibe was consistent with that predicted by a meta-analysis of randomized trials of statin therapy alone.^9^

Based on all randomized trial evidence, the Kidney Disease: Improving Global Outcomes (KDIGO) Clinical Practice Guideline for Lipid Management in CKD recommended use of a statin or statin/ezetimibe combination for adults ≥50 years old with an eGFR ≤60 ml/min per 1.73 m^2^ who are not on renal replacement therapy (RRT: chronic dialysis or kidney transplantation), and a statin alone for other adults with non-dialysis-dependent CKD.^10^ The 2018 ACC/AHA Multisociety Guideline on cholesterol management recommends initiating statin or statin/ezetimibe combination in ≥40-year-old CKD patients with an increased (>7.5%) 10-year risk of atherosclerotic cardiovascular disease.^11^, ^12^, ^13^ In the UK, the National Institute for Health and Care Excellence recommends atorvastatin 20 mg daily for the primary prevention of cardiovascular disease in people with eGFR <60 ml/min per 1.73 m^2^ and suggests considering a higher dose, and/or combination with ezetimibe, in more advanced CKD (e.g., eGFR <30 ml/min per 1.73 m^2^), for secondary cardiovascular disease prevention, or when the desired cholesterol reduction has not been achieved.^14^, ^15^

Despite these recommendations, it remains unclear which statin/ezetimibe combination is the most cost-effective treatment in moderate-to-advanced CKD (i.e., offers the greatest benefits and is affordable). A cost-effectiveness study of 5-year simvastatin plus ezetimibe treatment in SHARP concluded that high-intensity generic treatments, rather than more expensive proprietary treatments, are cost-effective.^16^ However, a more pertinent question for health care providers is the cost-effectiveness of long-term treatments. We used the SHARP CKD-CVD policy model^17^ to project lifetime risks of CVD and CKD progression and the net effects and cost-effectiveness of long-term statin/ezetimibe treatments in categories of patients with CKD.

## Results

### CKD patients

Results are presented only for nondialysis patients, since there is no clear evidence that LDL cholesterol-lowering therapy is effective in dialysis patients.^7^ The mean age of the 6235 patients in the SHARP study^6^, ^18^ was 63 years (SD, 12); 62% were male; 23% had diabetes; and 15% had a prior history of (noncoronary) vascular disease. There were 2020 participants with stage 3B disease; 2767 with stage 4 disease; and 1448 with stage 5 disease not on dialysis. The participants’ median 5-year cardiovascular risk ranged from 10% in those with stage 3B to 20% in nondialysis participants in stage 5, and from 6% to 31% across the 3 categories of baseline risk (Table 1). Within each stage of CKD, participants at higher risk at baseline were older and more likely to have previous vascular disease or diabetes (Supplementary Table S1).

**Table 1:** Characteristics of nondialysis SHARP participants by CKD stage and cardiovascular disease risk at baseline

	By CKD stage at baseline			By 5-year risk of cardiovascular disease at baseline		
	CKD stage 3B*	CKD stage 4	CKD stage 5, not on dialysis	Low (<10%)	Medium (10%-20%)	High (≥20%)
	n=2020	n=2767	n=1448	n=2151	n=2045	n=2039
Age, years	62 (11)	64 (12)	62 (12)	53 (8)	65 (9)	71 (9)
Male	1461 (72%)	1653 (60%)	760 (52%)	1080 (50%)	1337 (65%)	1457 (71%)
Current smoker	271 (13%)	336 (12%)	162 (11%)	219 (10%)	273 (13%)	277 (14%)
Previous vascular disease	283 (14%)	430 (16%)	217 (15%)	43 (2%)	146 (7%)	741 (36%)
Diabetes mellitus	469 (23%)	662 (24%)	293 (20%)	106 (5%)	345 (17%)	973 (48%)
Treated hypertension	1701 (84%)	2389 (86%)	1261 (87%)	1841 (86%)	1751 (85%)	1767 (87%)
Body-mass index, kg/m^2^	28 (5)	28 (6)	27 (5)	27 (5)	28 (5)	27 (6)
Diastolic blood pressure, mm Hg	80 (13)	79 (13)	80 (12)	82 (12)	80 (12)	77 (13)
Systolic blood pressure, mm Hg	139 (20)	139 (21)	141 (21)	132 (17)	139 (20)	147 (22)
LDL cholesterol, mmol/L	2.9 (0.8)	2.9 (0.8)	2.7 (0.9)	2.9 (0.8)	2.9 (0.9)	2.8 (0.9)
HDL cholesterol, mmol/L	1.1 (0.3)	1.1 (0.3)	1.1 (0.3)	1.2 (0.3)	1.1 (0.3)	1.1 (0.3)
Estimated 5-year risk of cardiovascular disease, median (IQR)	10% (6%, 18%)	14% (9%, 24%)	20% (11%, 32%)	6% (5%, 8%)	14% (12%, 17%)	31% (24%, 42%)
**CKD stage at baseline**						
CKD stage 3B^a^				967 (45%)	649 (32%)	404 (20%)
CKD stage 4				882 (41%)	968 (47%)	917 (45%)
CKD stage 5, not on dialysis				302 (14%)	428 (21%)	718 (35%)

CKD, chronic kidney disease; eGFR, estimated glomerular filtration rate; IQR, interquartile range; SHARP, Study of Heart and Renal Protection.

Results are shown as mean (SD) or N (%), as appropriate, unless otherwise specified. Ten participants on kidney transplant at baseline were excluded.

a 338 (17%) of participants with CKD stage 3A (eGFR 60-45 ml/min per 1.73 m^2^).

### Effects of statin/ezetimibe treatments

Of the treatments considered, the least potent was ezetimibe 10 mg daily, which reduced LDL cholesterol by 18.5%; the most potent was atorvastatin 40 mg plus ezetimibe 10 mg daily, which reduced LDL cholesterol by 60.1%. Atorvastatin 40 mg daily reduced LDL cholesterol by 48% (Supplementary Table S2). The proportional reductions in risk of major vascular events with use of ezetimibe 10 mg daily were 8% (99% confidence interval [CI], 2%, 14%) in stage 3B, 8% (–1%, 17%) in stage 4, and 8% (–1%, 16%) in stage 5 not on dialysis. The proportional reductions in risk of major vascular events with use of atorvastatin 40 mg daily were 20% (6%, 33%) in stage 3B, 20% (–3%, 38%) in stage 4, and 19% (–3%, 36%) in stage 5 not on dialysis. The proportional reductions in risk of major vascular events with use of atorvastatin 40 mg plus ezetimibe 10 mg daily were 25% (7%, 40%) in stage 3B, 25% (–4%, 45%) in stage 4, and 23% (–3%, 43%) in stage 5 not on dialysis (Supplementary Table S3).

### US cost-effectiveness of statin/ezetimibe treatments

In all categories of CKD patients, at current statin/ezetimibe prices (January 2019), treatment with ezetimibe 10 mg was both less effective and more expensive than treatment with atorvastatin 20 mg. Therefore, in health economic jargon, ezetimibe 10 mg was “dominated” by atorvastatin 20 mg; rosuvastatin 20 mg was dominated by similarly effective and slightly cheaper atorvastatin 40 mg; simvastatin 20 mg plus ezetimibe 10 mg was dominated by atorvastatin 40 mg; and atorvastatin 20 mg plus ezetimibe 10 mg was dominated by atorvastatin 40 mg (Supplementary Table S2). Additionally, atorvastatin 20 mg was projected to produce very similar health benefits at a similar additional cost per QALY to atorvastatin 40 mg (Supplementary Table S4). Therefore, we present results for atorvastatin 40 mg and atorvastatin 40 mg plus ezetimibe 10 mg only. However, since atorvastatin 20 mg could be considered a less intensive treatment option, and ezetimibe 10 mg could be used by patients who cannot tolerate or do not use a statin-based regimen, we also present results for these regimens.

Lifetime use of atorvastatin 40 mg is projected to increase life expectancy by 0.26 years (0.23 QALYs) at a net cost of $20,300/QALY in patients with stage 3B disease; 0.37 years (0.31 QALYs) at a net cost of $44,200/QALY in patients with stage 4 disease; and 0.31 years (0.26 QALYs) at $78,200/QALY in patients with stage 5 disease not on dialysis. Similarly, it would increase life expectancy by 0.29 years (0.26 QALYs) at $38,100/QALY in those at low cardiovascular risk (<10%); by 0.32 years (0.27 QALYs) at $41,000/QALY in those at medium cardiovascular risk (10%–20%); and by 0.36 years (0.29 QALYs) at $55,000/QALY in those at high cardiovascular risk (≥20%) (Table 2). Within each cardiovascular risk group, the net cost per QALY was lowest for patients in stage 3B and highest for those in stage 5 not on dialysis, whereas within each CKD stage, net costs per QALY were similar at all levels of cardiovascular risk (Supplementary Figure S1). In almost all subgroups, patients who were younger at treatment initiation were projected to benefit the most but at the highest net cost per QALY. For example, patients <60 years old were projected to gain between 0.28 QALYs (if at low risk) and 0.51 QALYs (if at high risk), at a net cost, respectively, of $42,000 to $76,400/QALY; whereas the respective estimates for the patients ≥70 years old were 0.13 (low risk) to 0.22 (high risk) QALYs, at a net cost, respectively, of $10,700 to $42,300/QALY (Supplementary Table S5).

**Table 2:** Health benefits and cost-effectiveness of statin-based treatments in moderate-to-advanced nondialysis CKD patients

Category of CKD patient	Atorvastatin 40 mg daily^a^ compared to no LDL-C lowering treatment			Ezetimibe 10 mg plus atorvastatin 40 mg daily compared to atorvastatin 40 mg daily		
	Life-years gained	QALYs gained	Additional cost per QALY^b^	Life-years gained	QALYs gained	Additional cost per QALY^b^
**(A) US health care setting**						
**By CKD stage at baseline**						
CKD stage 3B^c^	0.26	0.23	$20,300	0.06	0.05	$43,600
CKD stage 4	0.37	0.31	$44,200	0.08	0.07	$58,400
CKD stage 5, not on dialysis	0.31	0.26	$78,200	0.07	0.06	$91,500
**By 5-year risk of cardiovascular disease at baseline**						
Low (<10%)	0.29	0.26	$38,100	0.06	0.06	$65,100
Medium (10%-20%)	0.32	0.27	$41,000	0.07	0.06	$56,700
High (≥20%)	0.36	0.29	$55,000	0.08	0.07	$64,400
**(B) UK health care setting**						
**By CKD stage at baseline**						
CKD stage 3B^c^	0.28	0.25	£3800	0.07	0.06	£12,500
CKD stage 4	0.42	0.33	£10,500	0.09	0.07	£16,000
CKD stage 5, not on dialysis	0.37	0.29	£18,900	0.09	0.07	£23,900
**By 5-year risk of cardiovascular disease at baseline**						
Low (<10%)	0.33	0.29	£7,900	0.08	0.07	£17,800
Medium (10%-20%)	0.36	0.29	£9,400	0.08	0.07	£15,200
High (≥20%)	0.40	0.29	£14,200	0.09	0.07	£17,800

CKD, chronic kidney disease; eGFR, estimated glomerular filtration rate; LDL-C, low-density lipoprotein cholesterol; QALY, quality-adjusted life-year; UK, United Kingdom; US, United States.

The CKD and cardiovascular risk categories are derived directly from the 6235 moderate-to-advanced non–dialysis-dependent CKD patients in the Study of Heart and Renal Protection (SHARP).

a Atorvastatin 20 mg daily was projected to produce only slightly smaller health benefits at similar additional cost per QALY to atorvastatin 40 mg daily (see Supplementary Tables S4 and S7 for detailed results) and could be considered as an alternative less intensive treatment option.

b Costs and outcomes discounted at 3% per annum (US) or 3.5% per annum (UK).

c 338 (17%) of participants with CKD stage 3A (eGFR 60–45 ml/min per 1.73 m^2^).

**Figure 1:**
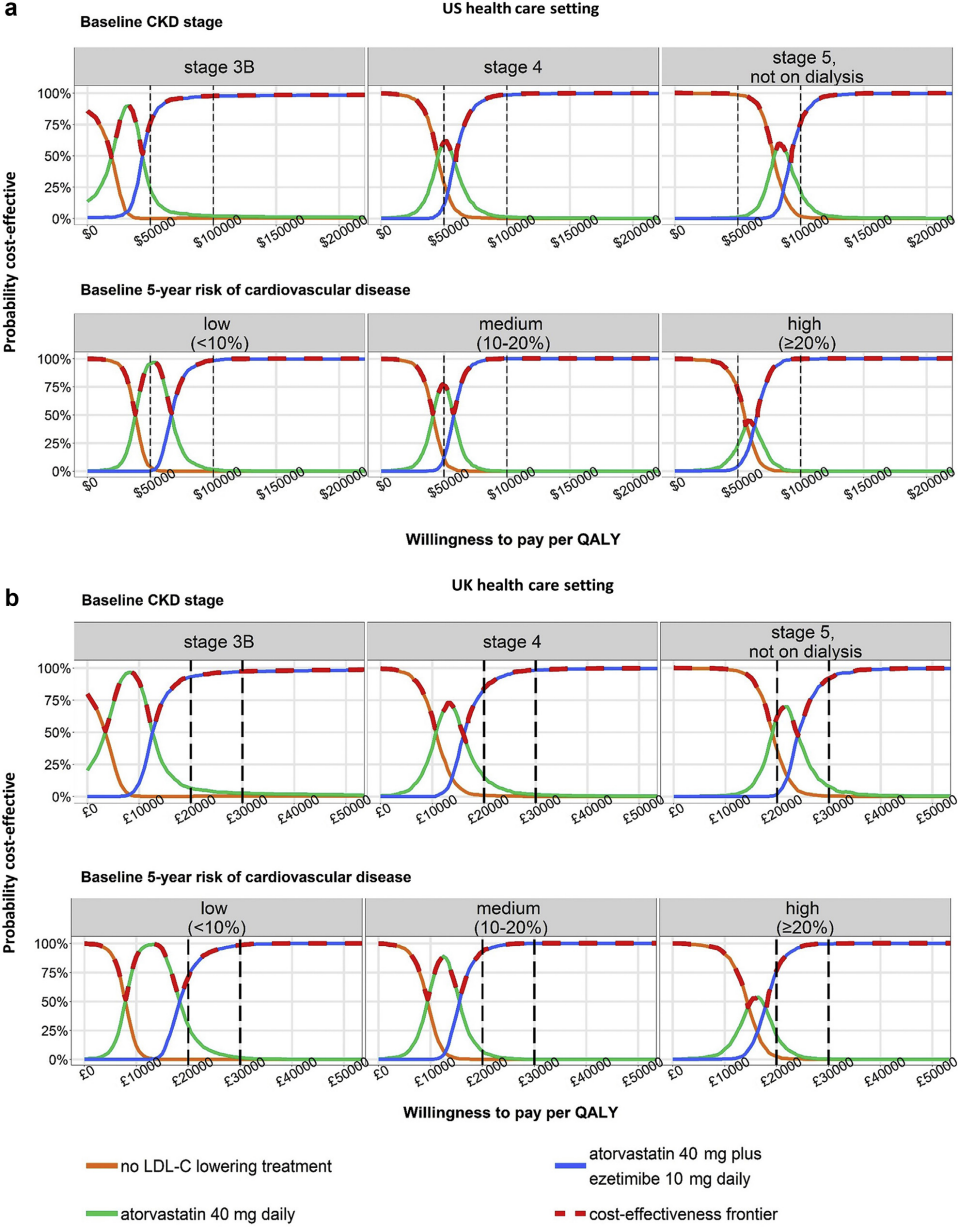
**Probability of a statin-based treatment to be cost-effective in moderate-to-advanced nondialysis chronic kidney disease (CKD) patients.** Results shown for treatments on the cost-effectiveness frontier (i.e., the most cost-effective treatment for a given value of willingness to pay) within the range of willingness-to-pay values per quality-adjusted life-year (QALY). Typical cost-effectiveness thresholds are represented with dashed horizontal lines. Atorvastatin 20 mg daily was largely dominated by atorvastatin 40 mg daily and was omitted from the graph. LDL-C, low-density lipoprotein cholesterol; UK, United Kingdom; US, United States.

Adding ezetimibe 10 mg was estimated to provide further benefits: there were an additional 0.06 years (0.05 QALYs, net cost $43,600/QALY) in stage 3B; 0.08 years (0.07 QALYs, net cost $58,400/QALY) in stage 4; and 0.07 years (0.06 QALYs, net cost $91,500/QALY) in stage 5 not on dialysis. There were an additional 0.06 years (0.06 QALYs, net cost $65,100/QALY) in low-risk patients; 0.07 years (0.06 QALYs, net cost $56,700/QALY) in medium-risk patients; and 0.08 years (0.07 QALYs, net cost $64,400/QALY) in high-risk patients (Table 2). At the $100,000/QALY cost-effectiveness threshold, atorvastatin 40 mg plus ezetimibe 10 mg would be considered cost-effective with a >95% probability in all patients except those in stage 5 not on dialysis (76%) (Figure 1).

Similar to treatment with atorvastatin 40 mg alone, the net cost per QALY with the combination of atorvastatin 40 mg and ezetimibe 10 mg daily, compared to atorvastatin 40 mg daily, was lowest for patients in stage 3B and highest for those in stage 5 not on dialysis, at each level of risk (Supplementary Figure S1).

Ezetimibe 10 mg daily, compared to no lipid-lowering treatment, was projected to result in 0.09 extra QALYs in stage 3B, 0.13 extra QALYs in stage 4, and 0.10 extra QALYs in stage 5 not on dialysis, at a net cost, respectively, of $31,000, $50,600, and $84,200/QALY, and 0.11 extra QALYs in low-risk patients, 0.11 extra QALYs in medium-risk patients, and 0.12 extra QALYs in high-risk patients at a net cost, respectively, of $50,900, $48,300, and $58,800/QALY (Supplementary Table S6).

### UK cost-effectiveness of statin/ezetimibe treatments

In the UK setting, ezetimibe is now also available at low prices from generic treatment manufacturers (£0.074/d, January 2019). The treatment benefits, cost-effectiveness, and policy implications for different statin/ezetimibe treatments (i.e., atorvastatin 40 mg daily and ezetimibe 10 mg daily alone and in combination with a statin) were similar to those in the US (Table 2, Figure 1, Supplementary Tables S5–S7, Supplementary Figure S1).

### Sensitivity analyses

**Figure 2:**
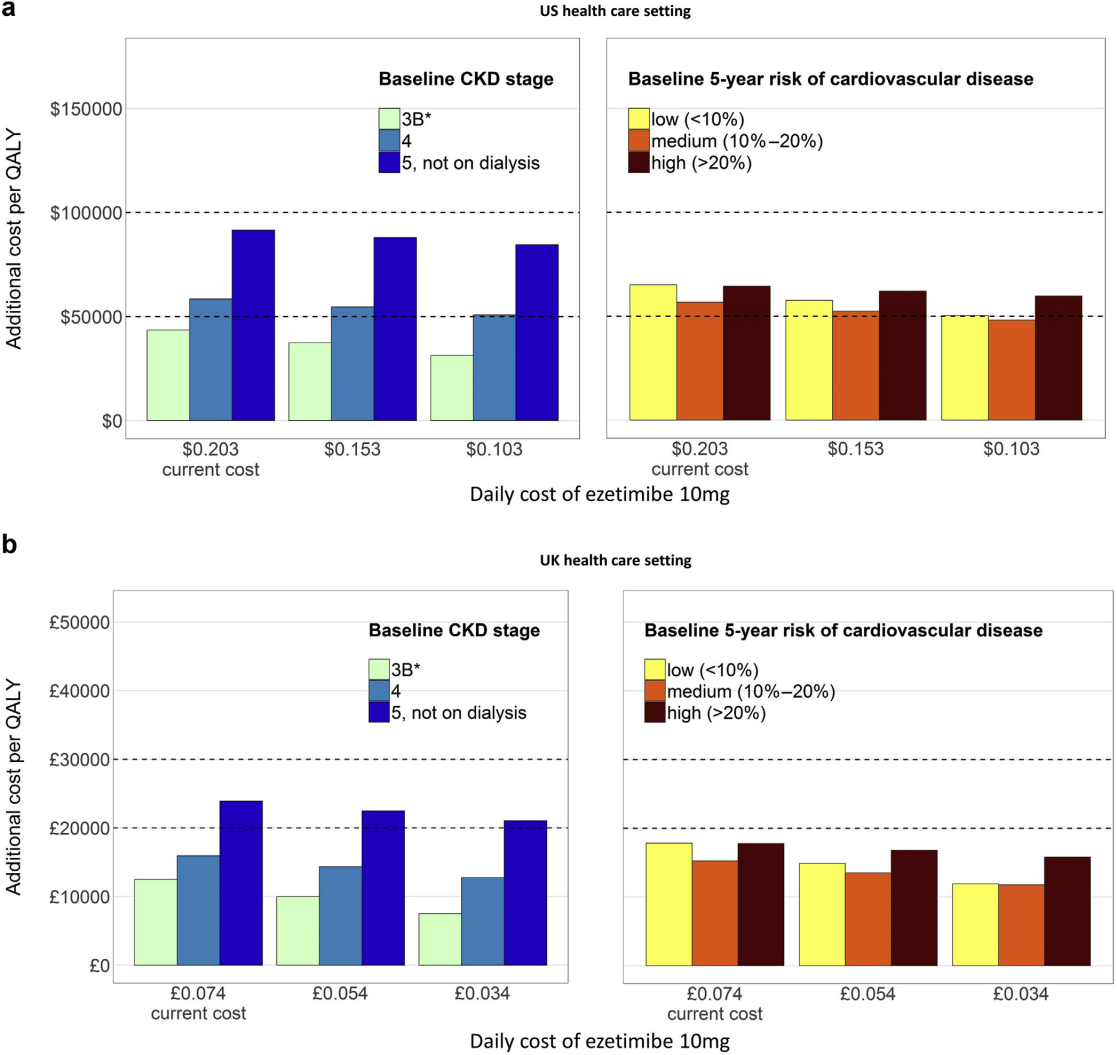
**Cost-effectiveness of adding ezetimibe 10 mg to atorvastatin 40 mg daily for moderate-to-advanced nondialysis chronic kidney disease (CKD) patients, at different ezetimibe cost.** The CKD and cardiovascular risk categories are derived directly from the 6235 moderate-to-advanced non–dialysis-dependent CKD patients in the Study of Heart and Renal Protection (SHARP). Typical cost-effectiveness thresholds are represented with dashed horizontal lines. *A total of 338 (17%) participants with CKD stage 3A (estimated glomerular filtration rate [eGFR] 60–45 ml/min per 1.73 m^2^). At the $100,000/quality-adjusted life-year [QALY] threshold in the United States (US) (**a**), ezetimibe 10 mg daily becomes cost-effective in all categories of patients when its price reaches $0.323/d. At the £20,000/QALY threshold in the United Kingdom (UK) (**b**), ezetimibe 10 mg daily becomes cost-effective in all categories of patients when its price reaches £0.019/d.

The cost-effectiveness results were only minimally sensitive to further falls in the price of ezetimibe (Figure 2). If ezetimibe 10 mg were priced at less than $0.323/d (£0.019/d in the UK), its net cost per QALY would be under $100,000 (£20,000) for all nondialysis CKD patients.

When analyses were repeated with the annual treatment costs for RRT assumed similar to those for CKD stage 5 not on dialysis, the net cost per QALY with statin/ezetimibe treatments decreased substantially. In the US setting, the net cost per QALY with atorvastatin 40 mg daily decreased from $20,300 to $5000 in stage 3B, from $44,200 to $6900 in stage 4, and from $78,200 to $7100 in CKD stage 5 not on dialysis. The net cost per QALY for atorvastatin 40 mg plus ezetimibe 10 mg daily decreased from $43,600 to $27,500 in stage 3B, from $58,400 to $20,600 in stage 4, and from $91,500 to $19,900 in stage 5 not on dialysis. The effect was similar in the UK setting (Supplementary Table S8).

The net costs per QALY only minimally increased when potential adverse effects of statin/ezetimibe treatments and their costs were projected (Supplementary Table S9). Reduced compliance with treatment was projected to result in lower health benefits but also lower incremental hospital costs and did not materially affect the results (Supplementary Table S10).

## Discussion

Lowering LDL cholesterol with statin-based treatments safely reduces cardiovascular risk in patients with moderate-to-advanced CKD who are not receiving maintenance dialysis, but there is no evidence that such treatment is effective in dialysis patients.^7^ We report that under the standard cost-effectiveness assumptions (i.e., a threshold of $100,000/QALY [£20,000 to £30,000/QALY in the UK]), low-cost statin treatments (e.g., atorvastatin 40 mg daily) are cost-effective in nondialysis CKD patients. Ezetimibe has recently come off patent in both countries and, at current prices ($0.203/d and £0.074/d, respectively), adding ezetimibe 10 mg to atorvastatin 40 mg daily is a cost-effective option in nondialysis CKD patients and would thus be the treatment of choice. The results remain robust across a range of sensitivity analyses.

Despite the higher net cost per QALY, the finding that low-cost generic statins/ezetimibe are cost-effective for primary prevention of CVD in CKD is consistent with results for the general population at an increased cardiovascular risk.^19^, ^20^ Unlike general population estimates, in non–dialysis-dependent CKD, the net cost per QALY was generally higher in patients at more advanced CKD stage and/or higher predicted cardiovascular risk, especially for very low-cost treatments (e.g., atorvastatin 40 mg or ezetimibe 10 mg at a lower price). This is driven by reduced life expectancy, higher end-stage kidney disease risks, and RRT costs, which are incurred during the gained life expectancy and are substantial in more advanced CKD. Statin-based treatments would also be more cost-effective in patients receiving a kidney transplant, with lower RRT costs in the years after the transplantation.

The present study builds upon a study by Erickson *et al.*^21^ and our findings support its conclusion that cheaper generic statins are likely cost-effective in CKD. However, our study takes a substantially more detailed look into the long-term effects of individual treatments across categories of CKD patients. We used the rich individual data from the SHARP study, which enabled us to present results generalizable to CKD patients at similar cardiovascular risk and/or CKD stage. We also used results from the CTT individual participant meta-analysis of 28 large trials, which provide the best available estimates for cardiovascular risk reductions with statin-based treatments at different levels of renal function.^7^ Linking these with the potency of individual statin/ezetimibe regimens enabled evaluation of the cost-effectiveness of different regimens reliably.

In the present study, treatment with rosuvastatin 20 mg was similarly effective but slightly more expensive than atorvastatin 40 mg (atorvastatin 40 mg is available at $0.103/d in the US and £0.034/d in the UK, compared to $0.119/d and £0.077/d, respectively, for rosuvastatin 20 mg). Small further fluctuations in prices would make rosuvastatin a cost-effective option. The use of less potent treatments (e.g., atorvastatin 20 mg or ezetimibe 10 mg alone) achieves smaller cardiovascular benefits. In general, statin/ezetimibe treatments that achieve larger reductions in LDL cholesterol are expected to achieve greater health benefits and, if available at low cost, be cost-effective. In the present analyses, we did not consider more potent statin treatments such as atorvastatin 80 mg and rosuvastatin 40 mg, since they are not routinely used in CKD patients due to safety concerns. However, if future evidence indicates safety of these regimens in CKD, our analysis suggests that, if available at low cost, they will be cost-effective.

Several limitations of the present analyses should be acknowledged. First, SHARP included only CKD patients without a prior history of myocardial infarction or coronary revascularization, whereas, in routine practice, coronary heart disease is highly prevalent in people with moderate-to-advanced CKD. Therefore, patients outside SHARP at similar CKD stages are likely to be at higher cardiovascular risk. However, the cost-effectiveness estimates corresponding to categories of risk are likely to be generalizable to such patients. Second, since SHARP did not directly assess the effects of ezetimibe monotherapy in patients with CKD, we incorporated an assumption that its effects on clinical outcomes are equivalent, per mmol/L reduction in LDL cholesterol, to those of statins. This assumption was derived from the results of the CTT meta-analysis of the effects of statin-based regimens at different levels of eGFR^7^ and by the IMPROVE-IT trial, which was conducted in people with an acute coronary syndrome treated with statin^8^ and demonstrated further cardiovascular risk reductions with ezetimibe similar to those with statin-only regimens achieving similar LDL cholesterol reductions.^9^ Third, the effect of treatments on non–health care costs, such as productivity or long-term care costs, were not included in the present analysis, which was conducted exclusively from a health services perspective. Finally, safety of statin-based regimens has been a subject of debate despite the acknowledgement that any adverse effects are rare and benefits strongly outweigh any harm.^22^, ^23^ We did not include adverse effects in our primary analysis due to the paucity of data for different treatments. However, sensitivity analyses incorporating estimated rates of potential adverse effects on muscle (e.g., myopathy and rhabdomyolysis) and diabetes yielded similar results.

In conclusion, statin-based treatments effectively reduce cardiovascular risk in nondialysis patients with CKD and, at current prices and cost-effectiveness thresholds, the available evidence suggests that low-cost statin/ezetimibe combination therapy is cost-effective. The most cost-effective regimen is one that maximizes the dose of statin chosen without compromising safety.

## Methods

### The SHARP CKD-CVD policy model

The SHARP CKD-CVD policy model, which is a Markov state-transition model developed using the SHARP study^6^, ^18^ data and validated in 3 external CKD cohorts,^17^ was used to project cardiovascular events, CKD progression, health care costs, and health-related quality of life (QoL). A full description of the model has been published elsewhere.^17^ Briefly, it is based on parametric risk equations (3 survival equations for cardiovascular outcomes, a multinomial regression and a logistic regression for CKD progression, and 2 linear regressions predicting hospital costs and QoL). Each equation includes a range of clinically and/or statistically important covariates, including the patient’s sociodemographic status, comorbidities, and risk factors (eg age, most recent CKD stage, and detailed cardiovascular disease history.

The model simulates annual risks of dying from vascular and nonvascular causes; experiencing a major atherosclerotic event or a hemorrhagic stroke; and progressing through CKD stages 3B (30 ≤ eGFR <45 ml/min per 1.73 m^2^), 4 (15 ≤ eGFR <30 ml/min per 1.73 m^2^), and 5 (eGFR <15 ml/min per 1.73 m^2^), or having dialysis or kidney transplant (Supplementary Figure S2).

### CKD patients

Results are presented only for the 6235 SHARP nondialysis patients (since previous analyses have shown that lowering LDL cholesterol is not clinically effective in dialysis patients^7^); all such patients had moderate-to-advanced CKD (Table 1, Supplementary Table S1). At baseline, the study participants were categorized according to their CKD stage: (i) CKD stage 3B; (ii) CKD stage 4; or (iii) CKD stage 5 not on dialysis. They were also categorized by their 5-year risk of major vascular event^16^: (i) low (<10%); (ii) medium (≥10%, <20%); and (iii) high (≥20%) risk. eGFR was calculated using the CKD-EPI equation.^24^ The rates of nonvascular mortality were obtained from relevant population data (see Supplementary Table S11 for US rates and Schlackow *et al.*^17^ for UK rates).

### Effects of statin/ezetimibe treatments

We considered a range of statin/ezetimibe regimens that are believed to be safe in CKD, including ezetimibe 10 mg, atorvastatin 20 mg, atorvastatin 40 mg, rosuvastatin 20 mg, simvastatin 20 mg plus ezetimibe 10 mg, atorvastatin 20 mg plus ezetimibe 10 mg, and atorvastatin 40 mg plus ezetimibe 10 mg daily. Treatment effects were projected on the risks of vascular death and major vascular events but not CKD progression^25^ or nonvascular mortality.^6^, ^7^ For each statin/ezetimibe regimen, the effects on vascular endpoints were expressed as relative risk reductions and evaluated separately for each CKD stage in 2 steps. First, the absolute reductions in LDL cholesterol were calculated using the expected proportional reductions in LDL cholesterol (Supplementary Table S2) and the mean LDL cholesterol of the SHARP participants in the respective CKD stage (Table 1, Supplementary Table S3). Second, these absolute reductions were combined with the rate ratios for vascular events per 1 mmol/L reduction in LDL cholesterol reported in the individual participant data meta-analysis by the CTT Collaboration (Supplementary Table S3).

### Costs and QoL

Annual hospital costs of managing a patient with CKD and cardiovascular complications were based on published data (see Supplementary Table S12 for the US costs and Kent *et al.*^26^ for the UK costs) and inflated to year 2015 using the Consumer Price Index^27^ (US) or Hospital & Community Health Services Index^28^ (UK). The costs of statin and ezetimibe treatments (January 2019) were obtained from the National Average Drug Acquisition Cost (NADAC) reports^29^ (US) and NHS Electronic Drug Tariff^30^ (UK) (Supplementary Table S2).

Patients’ health-related QoL was derived from responses to the EuroQoL 5-dimensions 3-level (EQ-5D-3L) questionnaire^31^ completed by participants at the final SHARP follow-up visit and using the US^32^ or UK^33^ EQ-5D-3L utility tariffs (see Supplementary Table S13 for US estimates and Schlackow *et al.*^17^ for UK estimates) and stratified by CKD stage, cardiovascular morbidity, and other characteristics.

### Cost-effectiveness analyses

The cost-effectiveness analyses were performed from the perspectives of the US and UK health care systems. Health outcomes and costs were projected with lifelong treatment with each statin/ezetimibe regimen as well as no treatment, until patients reached 95 years of age or died. Costs and QALYs were discounted at an annual rate of 3% (US^34^) or 3.5% (UK^35^). For each treatment, the incremental cost-effectiveness ratios were calculated as the incremental cost per QALY gained with the treatment against the next less effective (and not dominated) treatment.^36^ Results are presented for categories of participants by CKD stage and, separately, by cardiovascular risk at baseline. Uncertainty in the cost-effectiveness was assessed using the nonparametric bootstrap,^37^ with the analysis replicated on 1000 sets of risk, cost, and QoL parameter estimates derived from refitting the original SHARP CKD-CVD risk equations^17^ on bootstrapped SHARP data or, for US hospital costs, using sampled values from the parametric distribution of costs (Supplementary Table S12).^38^ Uncertainty in the treatment effects was incorporated using values sampled from the respective lognormal distribution corresponding to the relative risk (99% CI) reported by the CTT collaboration.^7^ Cost-effectiveness acceptability curves were derived to summarize the probability of each treatment being cost-effective at different levels of willingness-to-pay thresholds.^36^

A schematic of our approach is presented in Supplementary Figure S3.

### Sensitivity analyses

First, sensitivity analyses were performed to assess robustness of the results. The price of ezetimibe was varied from the current prices of $0.203/d (US) and £0.074/d (UK) to the price of atorvastatin 40 mg of $0.103/d (US) and £0.034/d (UK). The price at which ezetimibe becomes cost-effective for the commonly used thresholds of $100,000 (US) and £20,000 (UK) per QALY was calculated. Second, to estimate the likely effect of dialysis costs on cost-effectiveness results, the analyses were repeated with RRT costs replaced with those for CKD stage 5 not on dialysis. Third, an analysis incorporating potential rare adverse effects of atorvastatin 40 mg alone or in combination with ezetimibe 10 mg daily was performed. Specifically, we assumed that during each year in patients taking atorvastatin 40 mg daily (with or without ezetimibe), 0.011% will experience myopathy at a cost of $33 (£19; derived as the cost of 3 creatine kinase tests) and 0.001 QoL decrement (i.e., 0.017 decrement over 30 days), and 0.0042% will experience rhabdomyolysis at a cost of $13,600 (£8000), of whom 10% will die, with the rest experiencing a 3% QoL decrement (i.e., 50% decrement over 7.5 days of hospitalization followed by 20% decrement over 30 days of recovering) in the year of the rhabdomyolysis,^23^, ^39^ and 0.2% will develop diabetes.^40^ Finally, the effect of nonadherence to treatment was explored in scenarios where, respectively, 40%, 60%, and 80% of the patients were taking the medication.

All analyses were performed with R 3.4.1^41^; the graphs were produced with the ggplot2 plotting system.^42^

## Disclosure

The SHARP study, including the analyses presented here, was funded by Merck & Co., Inc., Kenilworth, NJ USA, with additional support from the British Heart Foundation (CH/1996001/9454), and the UK Medical Research Council (A310). SHARP was initiated, conducted, and interpreted independently of the principal study funder (Merck & Co.). WH is supported by a Medical Research Council and Kidney Research UK Professor David Kerr Clinician Scientist Award. BM and MJL are supported by the National Institute for Health Research (NIHR) Oxford Biomedical Research Centre (BRC). The study funders/sponsors did not have any role in study design; collection, analysis, and interpretation of data; writing the report; and the decision to submit the report for publication. The Clinical Trial Service Unit of the University of Oxford (Oxford, UK) has a staff policy of not accepting honoraria or other payments from the pharmaceutical industry, except for the reimbursement of costs to participate in scientific meetings. WH, JE, RH, RC, MJL, CB, and BM report other grants for unrelated work.

## Appendix

### The SHARP Collaborative Group steering committee

R. Collins (chair), C. Baigent (study coordinator and chief investigator), M.J. Landray (clinical coordinator and co-principal investigator), C. Bray, Y. Chen (administrative coordinators), A. Baxter, A. Young (computing coordinators), M. Hill (director, central laboratory), C. Knott (nursing coordinator), A. Cass, B. Feldt-Rasmussen, B. Fellström, D.E. Grobbee, C. Grönhagen-Riska, M. Haas, H. Holdaas, L.S. Hooi, L. Jiang, B. Kasiske, U. Krairittichai, A. Levin, Z.A. Massy, V. Tesar, R. Walker, C. Wanner, D.C. Wheeler, A. Wiecek (national coordinators), T. Dasgupta, W. Herrington, D. Lewis, M. Mafham, W. Majoni, C. Reith (clinical support), J. Emberson, S. Parish (statistics), D. Simpson (lay member), J. Strony, T. Musliner (Merck Schering Plough, nonvoting), L. Agodoa, J. Armitage, Z. Chen, J. Craig, D. de Zeeuw, J.M. Gaziano, R. Grimm, V. Krane, B. Neal, V. Ophascharoensuk, T. Pedersen, P. Sleight, J. Tobert, and C. Tomson. The full list of the SHARP investigators is available elsewhere.^6^

The procedure for access requests to the SHARP (Trial registration: NCT00125593) data is available at http://www.ndph.ox.ac.uk/about/data-access-policy. The SHARP CKD-CVD model interface and user guide are available at http://dismod.ndph.ox.ac.uk/kidneymodel/app/.

## Supplementary Material

Table S1 Characteristics of Study of Heart and Renal Protection (SHARP) nondialysis participants by chronic kidney disease (CKD) stage and cardiovascular disease risk at baseline.

Table S2 Reductions in low-density lipoprotein (LDL) cholesterol with statin-based treatments and daily drug treatment cost.

Table S3 Average low-density lipoprotein (LDL) cholesterol, relative risk with statin-based treatment per 1-mmol/L reduction in LDL cholesterol, and relative risk with specific statin-based treatments, by chronic kidney disease (CKD) stage.

Table S4 Health outcomes, United States (US) hospital care costs, and US additional cost per quality-adjusted life year with lifetime use of atorvastatin and ezetimibe treatments in moderate-to-advanced nondialysis chronic kidney disease (CKD).

Table S5 Cost-effectiveness of lifetime use of atorvastatin and ezetimibe treatments in categories of moderate-to-advanced nondialysis chronic kidney disease (CKD) patients, by age at treatment initiation.

Table S6 Added (quality-adjusted) life years, extra hospital care costs, and additional cost per quality-adjusted life year with lifetime use of ezetimibe 10 mg daily compared to no low-density lipoprotein cholesterol (LDL-C)-lowering treatment in moderate-to-advanced nondialysis chronic kidney disease (CKD).

Table S7 Health outcomes, United Kingdom (UK) hospital care costs, and UK additional cost per quality-adjusted life year with lifetime use of atorvastatin and ezetimibe treatments in moderate-to-advanced nondialysis chronic kidney disease (CKD).

Table S8 Sensitivity analysis of cost-effectiveness of lifetime use of atorvastatin and ezetimibe treatments in moderate-to-advanced non-dialysis chronic kidney disease (CKD) patients with future renal replacement therapy costs excluded.

Table S9 Sensitivity analysis of cost-effectiveness of lifetime use of atorvastatin and ezetimibe treatments in moderate-to-advanced non-dialysis chronic kidney disease (CKD) patients, incorporating potential adverse effects on myopathy, rhabdomyolysis, and diabetes.

Table S10 Sensitivity analysis of cost-effectiveness of lifetime use of atorvastatin and ezetimibe treatments in moderate-to-advanced non-dialysis chronic kidney disease (CKD) patients under different scenarios of compliance with treatment.

Table S11 Annual rates of nonvascular death in moderate-to-advanced chronic kidney disease (CKD) patients (United States).

Table S12 United States (US) annual hospital care costs in moderate-to-advanced chronic kidney disease (CKD).

Table S13 Health-related quality of life in moderate-to-advanced chronic kidney disease (CKD): a linear regression model derived from Study of Heart and Renal Protection (SHARP) participant data using United States (US) EQ-5D value set.

Figure S1 Additional quality-adjusted life-years (QALYs), hospital care costs, and cost-effectiveness of lifetime use of atorvastatin and ezetimibe treatments in categories of moderate-to-advanced nondialysis chronic kidney disease (CKD) patients, by CKD stage and cardiovascular risk at baseline.

Figure S2 Schematic of the Study of Heart and Renal Protection (SHARP) CKD-CVD lifetimes health outcomes model.

Figure S3 Schematic of the information sources of the Study of Heart and Renal Protection (SHARP) CKD-CVD lifetime health outcomes model.
