# Supplementary material for: Cost-effectiveness of lipid lowering with statins and ezetimibe in chronic kidney disease
Source: Kidney Int. 2019 Jul;96(1):170–9. doi: 10.1016/j.kint.2019.01.028 (PMC6595178; doi:10.1016/j.kint.2019.01.028)
Supplement: Table S1 — Characteristics of Study of Heart and Renal Protection (SHARP) nondialysis participants by chronic kidney disease (CKD) stage and cardiovascular disease risk at baseline. [file mmc1.pdf]

Table S1 Characteristics of Study of Heart and Renal Protection (SHARP) nondialysis participants by chronic kidney disease (CKD) stage and cardiovascular disease risk at baseline

By CKD stage at baseline	By 5-year risk of cardiovascular disease at baseline		
	Low (<10%)	Medium (10-20%)	High (≥20%)
**CKD stage 3B***			
**N**	**967**	**649**	**404**
Age, years	54 (9)	67 (9)	71 (9)
Male	607 (63%)	521 (80%)	333 (82%)
Current smoker	106 (11%)	100 (15%)	65 (16%)
Previous vascular disease	22 (2%)	76 (12%)	185 (46%)
Diabetes mellitus	61 (6%)	161 (25%)	247 (61%)
Treated hypertension	809 (84%)	547 (84%)	345 (85%)
Body-mass index, kg/m²	28 (5)	28 (5)	28 (5)
Diastolic blood pressure, mmHg	82 (12)	80 (12)	78 (14)
Systolic blood pressure, mmHg	133 (18)	141 (21)	147 (22)
LDL cholesterol, mmol/L	3.0 (0.8)	2.9 (0.8)	2.8 (0.9)
HDL cholesterol, mmol/L	1.2 (0.3)	1.1 (0.3)	1.0 (0.3)
**CKD stage 4**			
**N**	**882**	**968**	**917**
Age, years	53 (8)	66 (9)	73 (9)
Male	384 (44%)	600 (62%)	669 (73%)
Current smoker	88 (10%)	122 (13%)	126 (14%)
Previous vascular disease	16 (2%)	55 (6%)	359 (39%)
Diabetes mellitus	41 (5%)	159 (16%)	462 (50%)
Treated hypertension	765 (87%)	821 (85%)	803 (88%)
Body-mass index, kg/m²	27 (6)	28 (6)	28 (6)
Diastolic blood pressure, mmHg	82 (11)	80 (13)	77 (14)
Systolic blood pressure, mmHg	131 (16)	139 (21)	146 (22)
LDL cholesterol, mmol/L	3.0 (0.8)	2.9 (0.8)	2.9 (0.9)
HDL cholesterol, mmol/L	1.2 (0.3)	1.2 (0.3)	1.1 (0.3)
**CKD stage 5, not on dialysis**			
**N**	**302**	**428**	**718**
Age, years	49 (6)	60 (8)	70 (9)
Male	89 (29%)	216 (50%)	455 (63%)
Current smoker	25 (8%)	51 (12%)	86 (12%)
Previous vascular disease	5 (2%)	15 (4%)	197 (27%)
Diabetes mellitus	4 (1%)	25 (6%)	264 (37%)

Treated hypertension	266 (88%)	378 (88%)	617 (86%)
Body-mass index, kg/m²	26 (5)	27 (6)	27 (5)
Diastolic blood pressure, mmHg	82 (10)	81 (12)	78 (13)
Systolic blood pressure, mmHg	132 (17)	138 (18)	147 (22)
LDL cholesterol, mmol/L	2.7 (0.8)	2.7 (0.9)	2.7 (0.9)
HDL cholesterol, mmol/L	1.2 (0.3)	1.1 (0.3)	1.1 (0.3)

CKD, chronic kidney disease; HDL, high-density lipoprotein; IQR, interquartile range; LDL, low-density lipoprotein; SHARP, Study of Heart and Renal Protection.

Mean (standard deviation [SD]) or N (%) presented, as appropriate, unless otherwise specified. Ten participants on kidney transplant at baseline were excluded.

*338 (17%) of participants with CKD stage 3A (estimated glomerular filtration rate [eGFR] 60-45 mL/min/1.73 m^2).
