# Supplementary material for: Cost-effectiveness of lipid lowering with statins and ezetimibe in chronic kidney disease
Source: Kidney Int. 2019 Jul;96(1):170–9. doi: 10.1016/j.kint.2019.01.028 (PMC6595178; doi:10.1016/j.kint.2019.01.028)
Supplement: Table S2 — Reductions in low-density lipoprotein (LDL) cholesterol with statin-based treatments and daily drug treatment cost. [file mmc2.pdf]

Table S2 Reductions in low-density lipoprotein (LDL) cholesterol with statin-based treatments and daily drug treatment cost

Treatment and dose, mg/day	Reduction in LDL cholesterol	US daily cost, US $[a]	UK daily cost, UK £[b]
**Ezetimibe 10[c]**	18.5%	0.203	0.074
**Atorvastatin 20[d]**	44%	0.072	0.028
**Atorvastatin 40[d]**	48%	0.103	0.034
**Rosuvastatin 20[e]**	48%	0.119	0.077
**Simvastatin 20 plus ezetimibe 10[d]**	51%	2.031 (combined tablet)<br>0.233 (separate tablets)	1.194 (combined tablet)<br>0.101 (separate tablets)
**Atorvastatin 20 plus ezetimibe 10[d,f]**	56.1%	0.275 (separate tablets)	0.102 (separate tablets)
**Atorvastatin 40 plus ezetimibe 10[d,f]**	60.1%	0.306 (separate tablets)	0.108 (separate tablets)

UK, United Kingdom; US, United States

[a]National Average Drug Acquisition Cost (NADAC). Baltimore: Centers for Medicare & Medicaid Services; 2019. https://data.medicaid.gov/Drug-Pricing-and-Payment/NADAC-National-Average-Drug-Acquisition-Cost-/a4y5-998d. Accessed 2 January 2019.

[b]NHS Prescription Services. NHS Electronic Drug Tariff January 2019 https://www.nhsbsa.nhs.uk/sites/default/files/2018-12/Drug%20Tariff%20January%202019.pdf. Accessed 2 January 2019

[c]Knopp RH, Gitter H, Truitt T, et al. Effects of ezetimibe, a new cholesterol absorption inhibitor, on plasma lipids in patients with primary hypercholesterolemia. Eur Heart J. 2003;24(8):729-741.

[d]Ballantyne CM, Abate N, Yuan Z, King TR, Palmisano J. Dose-comparison study of the combination of ezetimibe and simvastatin (Vytorin) versus atorvastatin in patients with hypercholesterolemia: the Vytorin Versus Atorvastatin (VYVA) study. Am Heart J. 2005;149(3):464-73.

[e]National Institute for Health and Care Excellence. Lipid modification: cardiovascular risk assessment and the modification of blood lipids for the primary and secondary prevention of cardiovascular disease. National Institute for Health and Care Excellence; 2014.

[f]Ballantyne CM, Houri J, Notarbartolo A, et al. Effect of ezetimibe coadministered with atorvastatin in 628 patients with primary hypercholesterolemia: a prospective, randomized, double-blind trial. Circulation. 2003;107(19):2409-2415.
