# Supplementary material for: Cost-effectiveness of lipid lowering with statins and ezetimibe in chronic kidney disease
Source: Kidney Int. 2019 Jul;96(1):170–9. doi: 10.1016/j.kint.2019.01.028 (PMC6595178; doi:10.1016/j.kint.2019.01.028)
Supplement: Table S3 — Average low-density lipoprotein (LDL) cholesterol, relative risk with statin-based treatment per 1-mmol/L reduction in LDL cholesterol, and relative risk with specific statin-based treatments, by chronic kidney disease (CKD) stage. [file mmc3.pdf]

Table S3 Average low-density lipoprotein (LDL) cholesterol, relative risk with statin-based treatment per 1 mmol/L reduction in LDL cholesterol, and relative risk with specific statin-based treatments, by chronic kidney disease (CKD) stage

Treatment and dose, mg/day	CKD stage at baseline					
	CKD stage 3B	CKD stage 4	CKD stage 5, not on dialysis	CKD stage 3B	CKD stage 4	CKD stage 5, not on dialysis
**LDL cholesterol (SD) at baseline, mmol/L**	2.9 (0.8)	2.9 (0.8)	2.7 (0.9)	2.9 (0.8)	2.9 (0.8)	2.7 (0.9)
**Relative risk (99% CI) per 1 mmol/L reduction in LDL cholesterol with statin-based treatment[a]**	0.85 (0.75-0.96)	0.85 (0.71-1.02)	0.85 (0.71-1.02)	0.92 (0.81-1.05)	0.84 (0.70-1.00)	0.84 (0.70-1.00)
	**Rate ratio (99% CI) for major vascular event**			**Rate ratio (99% CI) for vascular death**		
**Ezetimibe 10**	0.92 (0.86-0.98)	0.92 (0.83-1.01)	0.92 (0.84-1.01)	0.96 (0.89-1.03)	0.91 (0.82-1.00)	0.92 (0.84-1.00)
**Atorvastatin 20**	0.81 (0.69-0.95)	0.81 (0.64-1.03)	0.82 (0.67-1.02)	0.90 (0.76-1.06)	0.80 (0.63-1.00)	0.81 (0.65-1.00)
**Atorvastatin 40**	0.80 (0.67-0.94)	0.80 (0.62-1.03)	0.81 (0.64-1.03)	0.89 (0.74-1.07)	0.78 (0.61-1.00)	0.80 (0.63-1.00)
**Rosuvastatin 20**	0.80 (0.67-0.94)	0.80 (0.62-1.03)	0.81 (0.64-1.03)	0.89 (0.74-1.07)	0.78 (0.61-1.00)	0.80 (0.63-1.00)
**Simvastatin 20 plus ezetimibe 10**	0.79 (0.65-0.94)	0.79 (0.60-1.03)	0.80 (0.62-1.03)	0.88 (0.73-1.08)	0.77 (0.59-1.00)	0.79 (0.61-1.00)
**Atorvastatin 20 plus ezetimibe 10**	0.77 (0.63-0.94)	0.77 (0.58-1.03)	0.79 (0.61-1.03)	0.88 (0.72-1.08)	0.76 (0.57-1.00)	0.78 (0.59-1.00)
**Atorvastatin 40 plus ezetimibe 10**	0.75 (0.60-0.93)	0.75 (0.55-1.04)	0.77 (0.57-1.03)	0.86 (0.69-1.09)	0.74 (0.53-1.00)	0.75 (0.56-1.00)

SD, standard deviation

Rate ratios were calculated using the absolute reduction in low-density lipoprotein cholesterol (LDL-C) achieved with treatment, based on the mean baseline LDL-C within the respective category of Study of Heart and Renal Protection (SHARP) participants (presented in first data row above), the proportional

reduction with treatment (Table S2), and rate ratios (99% confidence intervals [CI]) per 1 mmol/L reduction in LDL-C with statin-based therapy reported by the Cholesterol Treatment Trialists' (CTT) Collaboration (presented in second data row above). For example, the ezetimibe 10 relative risk in CKD stage 3B for major vascular event is calculated as exp(2.92*0.185*log(0.85)), where 2.92 is the mean baseline LDL-C across SHARP participants in CKD stage 3B; 0.185 is the proportional reduction achieved with ezetimibe 10mg/day (ie. 18.5%), and 0.85 is the relative risk for major vascular event per 1 mmol/L reduction in stage CKD 3B. The effect on major atherosclerotic events and vascular death was obtained by applying the scaling of 1.03 to the effect on major vascular events, to reflect the ratio of the two corresponding treatment effects observed in SHARP (respectively, -0.201 and -0.195 on the log scale).

[a]Cholesterol Treatment Trialists' Collaboration, Impact of renal function on the effects of LDL cholesterol lowering with statin-based treatments: a meta-analysis of individual participant data from 28 randomised trials. Lancet Diabetes Endocrinol. 2016;4(10):829-839.
