# Supplementary material for: Cost-effectiveness of lipid lowering with statins and ezetimibe in chronic kidney disease
Source: Kidney Int. 2019 Jul;96(1):170–9. doi: 10.1016/j.kint.2019.01.028 (PMC6595178; doi:10.1016/j.kint.2019.01.028)
Supplement: Table S5 — Cost-effectiveness of lifetime use of atorvastatin and ezetimibe treatments in categories of moderate-to-advanced nondialysis chronic kidney disease (CKD) patients, by age at treatment initiation. [file mmc5.pdf]

**Table S5 Cost-effectiveness of lifetime use of atorvastatin and ezetimibe treatments in categories of moderate-to-advanced nondialysis chronic kidney disease (CKD) patients, by age at treatment initiation**

| Age, years | <60 | | | 60-70 | | | ≥70 | | | All patients | | |
|---|---|---|---|---|---|---|---|---|---|---|---|---|
| | Life-years gained | QALYs gained | Additional cost per QALY[a] | Life-years gained | QALYs gained | Additional cost per QALY[a] | Life-years gained | QALYs gained | Additional cost per QALY[a] | Life-years gained | QALYs gained | Additional cost per QALY[a] |
| **A. US healthcare setting** | | | | | | | | | | | | |
| **Atorvastatin 40mg daily[b] compared to no LDL-C lowering treatment** | | | | | | | | | | | | |
| **By CKD stage at baseline** | | | | | | | | | | | | |
| CKD stage 3B[c] | 0.29 | 0.27 | $27,700 | 0.27 | 0.24 | $17,000 | 0.19 | 0.17 | $9,100 | 0.26 | 0.23 | $20,300 |
| CKD stage 4 | 0.44 | 0.38 | $53,300 | 0.39 | 0.32 | $44,700 | 0.28 | 0.23 | $31,300 | 0.37 | 0.31 | $44,200 |
| CKD stage 5, not on dialysis | 0.37 | 0.32 | $77,000 | 0.30 | 0.24 | $81,800 | 0.22 | 0.18 | $76,700 | 0.31 | 0.26 | $78,200 |
| **By 5-year risk of cardiovascular disease at baseline** | | | | | | | | | | | | |
| Low (<10%) | 0.31 | 0.28 | $42,000 | 0.22 | 0.20 | $20,500 | 0.14 | 0.13 | $10,700 | 0.29 | 0.26 | $38,100 |
| Medium (10-20%) | 0.46 | 0.40 | $56,000 | 0.31 | 0.27 | $36,700 | 0.19 | 0.17 | $20,000 | 0.32 | 0.27 | $41,000 |
| High (≥20%) | 0.62 | 0.51 | $76,400 | 0.43 | 0.35 | $60,700 | 0.27 | 0.22 | $42,300 | 0.36 | 0.29 | $55,000 |
| **Ezetimibe 10mg plus atorvastatin 40mg daily compared to atorvastatin 40mg daily** | | | | | | | | | | | | |
| **By CKD stage at baseline** | | | | | | | | | | | | |
| CKD stage 3B[c] | 0.07 | 0.06 | $55,000 | 0.06 | 0.06 | $37,300 | 0.04 | 0.04 | $28,000 | 0.06 | 0.05 | $43,600 |
| CKD stage 4 | 0.10 | 0.09 | $70,200 | 0.09 | 0.07 | $57,700 | 0.06 | 0.05 | $43,300 | 0.08 | 0.07 | $58,400 |
| CKD stage 5, not on dialysis | 0.09 | 0.07 | $92,600 | 0.07 | 0.06 | $93,500 | 0.05 | 0.04 | $87,100 | 0.07 | 0.06 | $91,500 |

| | | | | | | | | | | | | |
|---|---|---|---|---|---|---|---|---|---|---|---|---|
| **By 5-year risk of cardiovascular disease at baseline** | | | | | | | | | | | | |
| Low (<10%) | 0.07 | 0.06 | $68,700 | 0.05 | 0.04 | $48,100 | 0.03 | 0.03 | $43,200 | 0.06 | 0.06 | $65,100 |
| Medium (10-20%) | 0.10 | 0.09 | $69,500 | 0.07 | 0.06 | $52,500 | 0.04 | 0.04 | $39,400 | 0.07 | 0.06 | $56,700 |
| High (≥20%) | 0.15 | 0.12 | $83,900 | 0.10 | 0.08 | $69,500 | 0.06 | 0.05 | $52,600 | 0.08 | 0.07 | $64,400 |
| **B. UK healthcare setting** | | | | | | | | | | | | |
| **Atorvastatin 40mg daily[b] compared to no LDL-C lowering treatment** | | | | | | | | | | | | |
| **By CKD stage at baseline** | | | | | | | | | | | | |
| CKD stage 3B[c] | 0.33 | 0.30 | £5,700 | 0.29 | 0.25 | £3,200 | 0.19 | 0.16 | £600 | 0.28 | 0.25 | £3,800 |
| CKD stage 4 | 0.52 | 0.43 | £12,100 | 0.44 | 0.34 | £11,300 | 0.29 | 0.22 | £7,300 | 0.42 | 0.33 | £10,500 |
| CKD stage 5, not on dialysis | 0.46 | 0.37 | £17,200 | 0.36 | 0.27 | £21,000 | 0.25 | 0.18 | £20,400 | 0.37 | 0.29 | £18,900 |
| **By 5-year risk of cardiovascular disease at baseline** | | | | | | | | | | | | |
| Low (<10%) | 0.36 | 0.32 | £8,700 | 0.24 | 0.21 | £4,100 | 0.15 | 0.13 | £1,200 | 0.33 | 0.29 | £7,900 |
| Medium (10-20%) | 0.54 | 0.44 | £12,600 | 0.35 | 0.28 | £8,700 | 0.20 | 0.17 | £3,900 | 0.36 | 0.29 | £9,400 |
| High (≥20%) | 0.71 | 0.53 | £19,200 | 0.49 | 0.36 | £16,000 | 0.29 | 0.21 | £10,700 | 0.40 | 0.29 | £14,200 |
| **Ezetimibe 10mg plus atorvastatin 40mg daily compared to atorvastatin 40mg daily** | | | | | | | | | | | | |
| **By CKD stage at baseline** | | | | | | | | | | | | |
| CKD stage 3B[c] | 0.08 | 0.07 | £15,700 | 0.07 | 0.06 | £10,800 | 0.04 | 0.04 | £7,800 | 0.07 | 0.06 | £12,500 |
| CKD stage 4 | 0.12 | 0.10 | £18,400 | 0.10 | 0.08 | £16,200 | 0.07 | 0.05 | £12,000 | 0.09 | 0.07 | £16,000 |
| CKD stage 5, not on dialysis | 0.11 | 0.09 | £23,000 | 0.08 | 0.06 | £25,400 | 0.06 | 0.04 | £24,400 | 0.09 | 0.07 | £23,900 |
| **By 5-year risk of cardiovascular disease at baseline** | | | | | | | | | | | | |
| Low (<10%) | 0.08 | 0.07 | £18,500 | 0.05 | 0.05 | £14,500 | 0.03 | 0.03 | £13,600 | 0.08 | 0.07 | £17,800 |

| | | | | | | | | | | | | |
|---|---|---|---|---|---|---|---|---|---|---|---|---|
| Medium (10-20%) | 0.12 | 0.10 | £17,600 | 0.08 | 0.06 | £14,700 | 0.04 | 0.04 | £11,400 | 0.08 | 0.07 | £15,200 |
| High (≥20%) | 0.17 | 0.13 | £22,000 | 0.12 | 0.08 | £19,400 | 0.07 | 0.05 | £14,700 | 0.09 | 0.07 | £17,800 |

LDL-C, low-density lipoprotein cholesterol; QALY, quality-adjusted life-year; UK, United Kingdom; US, United States.

The analyses use localised non-vascular mortality rates and the utilities' value set.

[a]Costs and outcomes discounted at 3% per annum (US) and at 3.5% per annum (UK).

[b]Atorvastatin 20 mg daily was projected to produce only slightly smaller health benefits at similar additional cost per QALY to atorvastatin 40 mg daily (see Supplementary Tables S4 and S7 for detailed results) and could be considered as an alternative less intensive treatment option.

[c]338 (17%) of participants with CKD stage 3A (estimated glomerular filtration rate [eGFR] 60-45 mL/min/1.73 $m^2$).
