# Supplementary material for: Cost-effectiveness of lipid lowering with statins and ezetimibe in chronic kidney disease
Source: Kidney Int. 2019 Jul;96(1):170–9. doi: 10.1016/j.kint.2019.01.028 (PMC6595178; doi:10.1016/j.kint.2019.01.028)
Supplement: Table S6 — Added (quality-adjusted) life years, extra hospital care costs, and additional cost per quality-adjusted life year with lifetime use of ezetimibe 10 mg daily compared to no low-density lipoprotein cholesterol (LDL-C)-lowering treatment in moderate-to-advanced nondialysis chronic kidney disease (CKD). [file mmc6.pdf]

Table S6 Added (quality-adjusted) life years, extra hospital care costs, and additional cost per quality-adjusted life year with lifetime use of ezetimibe 10 mg daily compared to no low-density lipoprotein cholesterol (LDL-C)-lowering treatment in moderate-to-advanced nondialysis chronic kidney disease (CKD)

Category of CKD patient	Life-years gained (95% CI)	QALYs gained (95% CI)	Additional hospital care costs (95% CI)	Additional cost per QALY[a] (95% CI)
**A. US healthcare setting**				
**By CKD stage at baseline**				
CKD stage 3B[b]	0.10 (0.00, 0.19)	0.09 (0.01, 0.17)	$1,900 (-$1,500, $4,900)	$31,000 (-$12,000, $48,900)
CKD stage 4	0.15 (0.05, 0.25)	0.13 (0.05, 0.21)	$5,600 ($1,100, $10,000)	$50,600 ($32,100, $76,500)
CKD stage 5, not on dialysis	0.12 (0.04, 0.21)	0.10 (0.03, 0.17)	$7,800 ($1,900, $13,400)	$84,200 ($65,700, $109,200)
**By 5-year risk of cardiovascular disease at baseline**				
Low (<10%)	0.12 (0.05, 0.18)	0.11 (0.04, 0.16)	$3,800 ($900, $6,500)	$50,900 ($38,100, $70,300)
Medium (10-20%)	0.13 (0.05, 0.20)	0.11 (0.05, 0.17)	$4,500 ($1,000, $7,800)	$48,300 ($32,900, $66,400)
High (≥20%)	0.14 (0.05, 0.22)	0.12 (0.05, 0.18)	$6,400 ($1,600, $10,800)	$58,800 ($37,300, $78,200)
**B. UK healthcare setting**				
**By CKD stage at baseline**				
CKD stage 3B[b]	0.11 (0.00, 0.21)	0.10 (0.02, 0.17)	£400 (-£500, £1,100)	£7,900 (-£2,600, £13,800)
CKD stage 4	0.17 (0.05, 0.28)	0.13 (0.05, 0.21)	£1,400 (£200, £2,500)	£13,000 (£7,700, £21,500)
CKD stage 5, not on dialysis	0.15 (0.04, 0.25)	0.12 (0.04, 0.19)	£2,000 (£500, £3,500)	£21,200 (£16,300, £29,900)

By 5-year risk of cardiovascular disease at baseline				
Low (<10%)	0.14<br>(0.05, 0.21)	0.12<br>(0.05, 0.18)	£900<br>(£100, £1,500)	£12,700<br>(£9,200, £18,900)
Medium (10-20%)	0.14<br>(0.06, 0.22)	0.12<br>(0.05, 0.18)	£1,100<br>(£200, £1,900)	£12,100<br>(£8,200, £18,000)
High (≥20%)	0.16<br>(0.06, 0.25)	0.12<br>(0.05, 0.18)	£1,700<br>(£400, £2,900)	£15,700<br>(£9,700, £22,000)

CI, confidence interval; LDL-C, low-density lipoprotein cholesterol; QALY, quality-adjusted life-year; UK, United Kingdom; US, United States

[a]Costs and effects discounted at 3% per annum (US) or 3.5% per annum (UK).

[b]338 (17%) of participants with CKD stage 3A (estimated glomerular filtration rate [eGFR] 60-45 mL/min/1.73 m^2).
