# Supplementary material for: Cost-effectiveness of lipid lowering with statins and ezetimibe in chronic kidney disease
Source: Kidney Int. 2019 Jul;96(1):170–9. doi: 10.1016/j.kint.2019.01.028 (PMC6595178; doi:10.1016/j.kint.2019.01.028)
Supplement: Table S7 — Health outcomes, United Kingdom (UK) hospital care costs, and UK additional cost per quality-adjusted life year with lifetime use of atorvastatin and ezetimibe treatments in moderate-to-advanced nondialysis chronic kidney disease (CKD). [file mmc7.pdf]

Table S7 Health outcomes, United Kingdom (UK) hospital care costs, and UK additional cost per quality-adjusted life year with lifetime use of atorvastatin and ezetimibe treatments in moderate-to-advanced nondialysis chronic kidney disease (CKD)

	Absolute benefits			Incremental benefits (cost-effectiveness frontier)			
**Category of CKD patient**	**Life expectancy, years (95% CI)**	**Quality-adjusted life expectancy, QALYs (95% CI)**	**Hospital care costs (95% CI)**	**Life-years gained (95% CI)**	**QALYs gained (95% CI)**	**Additional hospital care costs (95% CI)**	**Additional cost (UK £) per QALY[a] (95% CI)**
**No LDL-C lowering treatment**							
**By CKD stage at baseline**							
CKD stage 3B[b]	15.98 (15.64, 16.34)	12.73 (12.45, 13.03)	£60,100 (£56,600, £63,800)	n/a	n/a	n/a	n/a
CKD stage 4	13.12 (12.84, 13.42)	10.23 (10.00, 10.48)	£77,900 (£73,700, £81,900)	n/a	n/a	n/a	n/a
CKD stage 5, not on dialysis	10.96 (10.54, 11.39)	8.59 (8.27, 8.93)	£121,700 (£115,400, £127,900)	n/a	n/a	n/a	n/a
**By 5-year risk of cardiovascular disease at baseline**							
Low (<10%)	20.11 (19.69, 20.50)	16.49 (16.14, 16.84)	£107,900 (£102,300, £112,900)	n/a	n/a	n/a	n/a
Medium (10-20%)	12.57 (12.26, 12.86)	9.74 (9.48, 9.97)	£76,500 (£72,500, £80,300)	n/a	n/a	n/a	n/a
High (≥20%)	7.61 (7.33, 7.91)	5.46 (5.23, 5.68)	£61,000 (£56,800, £65,800)	n/a	n/a	n/a	n/a
**Atorvastatin 20mg daily**				**Atorvastatin 20mg daily compared to no LDL-C lowering treatment**			
**By CKD stage at baseline**							
CKD stage 3B[b]	16.23 (15.81, 16.59)	12.96 (12.63, 13.26)	£61,000 (£56,700, £64,600)	0.26 (0.00, 0.48)	0.23 (0.04, 0.39)	£900 (-£1,100, £2,600)	£3,700 (-£13,800, £8,100)
CKD stage 4	13.50 (13.13, 13.86)	10.54 (10.23, 10.81)	£81,000 (£76,300, £85,500)	0.38 (0.12, 0.62)	0.30 (0.11, 0.48)	£3,100 (£500, £5,600)	£10,400 (£3,600, £16,900)
CKD stage 5, not on dialysis	11.30 (10.83, 11.74)	8.86 (8.51, 9.19)	£126,400 (£119,400, £133,300)	0.34 (0.10, 0.56)	0.27 (0.10, 0.43)	£4,700 (£1,100, £8,000)	£18,800 (£12,300, £25,200)

**By 5-year risk of cardiovascular disease at baseline**							
Low (<10%)	20.41 (19.99, 20.79)	16.75 (16.39, 17.07)	£109,900 (£104,200, £115,000)	0.31 (0.12, 0.47)	0.27 (0.12, 0.40)	£2,000 (£400, £3,400)	£7,800 (£3,000, £11,400)
Medium (10-20%)	12.88 (12.53, 13.19)	10.00 (9.72, 10.25)	£79,100 (£74,700, £83,100)	0.33 (0.13, 0.50)	0.27 (0.12, 0.40)	£2,500 (£500, £4,300)	£9,300 (£3,800, £13,900)
High (≥20%)	7.98 (7.62, 8.31)	5.73 (5.44, 5.97)	£65,000 (£59,900, £70,300)	0.37 (0.14, 0.55)	0.27 (0.11, 0.40)	£3,900 (£900, £6,600)	£14,100 (£6,500, £20,100)
**Atorvastatin 40mg daily**				**Atorvastatin 40mg daily compared to atorvastatin 20mg daily**			
**By CKD stage at baseline**							
CKD stage 3B[b]	16.26 (15.83, 16.63)	12.98 (12.63, 13.28)	£61,100 (£56,700, £64,800)	0.02 (0.00, 0.04)	0.02 (0.00, 0.03)	£100 (-£100, £200)	£5,400 (-£11,100, £9,600)
CKD stage 4	13.54 (13.15, 13.90)	10.56 (10.24, 10.84)	£81,300 (£76,500, £85,900)	0.03 (0.01, 0.05)	0.03 (0.01, 0.04)	£300 (£, £500)	£11,400 (£5,600, £18,500)
CKD stage 5, not on dialysis	11.33 (10.85, 11.77)	8.88 (8.52, 9.22)	£126,800 (£119,600, £133,700)	0.03 (0.01, 0.05)	0.02 (0.01, 0.03)	£400 (£100, £700)	£19,800 (£14,500, £26,700)
**By 5-year risk of cardiovascular disease at baseline**							
Low (<10%)	20.44 (20.01, 20.82)	16.77 (16.41, 17.10)	£110,100 (£104,300, £115,200)	0.03 (0.01, 0.04)	0.02 (0.01, 0.03)	£200 (£, £300)	£9,500 (£6,000, £13,500)
Medium (10-20%)	12.91 (12.55, 13.22)	10.02 (9.74, 10.28)	£79,300 (£74,900, £83,400)	0.03 (0.01, 0.04)	0.02 (0.01, 0.03)	£200 (£, £300)	£10,300 (£5,800, £15,100)
High (≥20%)	8.01 (7.64, 8.35)	5.75 (5.45, 6.00)	£65,300 (£60,100, £70,700)	0.03 (0.01, 0.05)	0.02 (0.01, 0.03)	£300 (£100, £600)	£14,900 (£8,000, £21,100)
**Atorvastatin 40mg plus ezetimibe 10mg daily**				**Atorvastatin 40mg plus ezetimibe 10mg compared to atorvastatin 40mg daily**			
**By CKD stage at baseline**							
CKD stage 3B[b]	16.32 (15.84, 16.72)	13.04 (12.65, 13.35)	£61,300 (£56,700, £65,100)	0.07 (0.00, 0.12)	0.06 (0.01, 0.09)	£300 (-£300, £700)	£12,500 (£8,100, £27,100)
CKD stage 4	13.63 (13.19, 14.02)	10.63 (10.28, 10.93)	£82,100 (£76,900, £86,900)	0.09 (0.03, 0.14)	0.07 (0.03, 0.11)	£800 (£100, £1,300)	£16,000 (£11,600, £27,700)
CKD stage 5, not on dialysis	11.42 (10.90, 11.87)	8.95 (8.56, 9.30)	£128,000 (£120,400, £135,300)	0.09 (0.03, 0.13)	0.07 (0.02, 0.10)	£1,200 (£300, £1,900)	£23,900 (£20,200, £35,000)
**By 5-year risk of cardiovascular disease at baseline**							
Low (<10%)	20.51 (20.07, 20.89)	16.84 (16.45, 17.17)	£110,600 (£104,800, £115,700)	0.08 (0.03, 0.11)	0.07 (0.03, 0.09)	£500 (£100, £800)	£17,800 (£14,500, £28,500)

Medium (10-20%)	12.99 (12.61, 13.32)	10.09 (9.79, 10.35)	£79,900 (£75,300, £84,100)	0.08 (0.03, 0.11)	0.07 (0.03, 0.09)	£600 (£100, £1,000)	£15,200 (£12,200, £22,700)
High (≥20%)	8.10 (7.70, 8.45)	5.82 (5.51, 6.09)	£66,300 (£60,700, £72,000)	0.09 (0.04, 0.13)	0.07 (0.03, 0.10)	£1,000 (£200, £1,700)	£17,800 (£13,300, £25,000)

LDL-C, low-density lipoprotein cholesterol; n/a, not applicable QALY, quality-adjusted life-year.

The analysis uses UK non-vascular mortality rates and the UK EQ-5D utilities' value set. In all patient subgroups, rosuvastatin 20mg was dominated by cheaper, and with the same effectiveness, atorvastatin 40mg; simvastatin 20mg plus ezetimibe 10mg was extendedly dominated by atorvastatin 40mg and atorvastatin 20mg plus ezetimibe 10mg; and atorvastatin 20mg plus ezetimibe 10mg was extendedly dominated by atorvastatin 40mg and atorvastatin 40mg plus ezetimibe 10mg. The interventions therefore are excluded from the table. Ezetimibe 10mg, whilst dominated by the cheaper and more effective atorvastatin 20mg, may be an option for CKD patients who cannot use statin, and therefore, the cost-effectiveness results for ezetimibe versus no LDL cholesterol lowering treatment are included in Supplementary Table S6.

[a]Costs and effects discounted at 3.5% per annum.

[b]338 (17%) of participants with CKD stage 3A (estimated glomerular filtration rate [eGFR] 60-45 mL/min/1.73 m^2).
