# Supplementary material for: Cost-effectiveness of lipid lowering with statins and ezetimibe in chronic kidney disease
Source: Kidney Int. 2019 Jul;96(1):170–9. doi: 10.1016/j.kint.2019.01.028 (PMC6595178; doi:10.1016/j.kint.2019.01.028)
Supplement: Table S9 — Sensitivity analysis of cost-effectiveness of lifetime use of atorvastatin and ezetimibe treatments in moderate-to-advanced non-dialysis chronic kidney disease (CKD) patients, incorporating potential adverse effects on myopathy, rhabdomyolysis, and diabetes. [file mmc9.pdf]

Table S9 Sensitivity analysis of cost-effectiveness of lifetime use of atorvastatin and ezetimibe treatments in moderate-to-advanced nondialysis chronic kidney disease (CKD) patients, incorporating potential adverse effects on myopathy, rhabdomyolysis, and diabetes

Category of CKD patient	Atorvastatin 40mg daily[a] compared to no LDL-C lowering treatment			Ezetimibe 10mg plus atorvastatin 40mg daily compared to atorvastatin 40mg daily		
	Life-years gained	QALYs gained	Additional cost per QALY[b]	Life-years gained	QALYs gained	Additional cost per QALY[b]
**A. US healthcare setting**						
**By CKD stage at baseline**						
CKD stage 3B[c]	0.26	0.23	$21,100	0.06	0.05	$43,600
CKD stage 4	0.37	0.31	$44,800	0.08	0.07	$58,400
CKD stage 5, not on dialysis	0.31	0.26	$78,900	0.07	0.06	$91,600
**By 5-year risk of cardiovascular disease at baseline**						
Low (<10%)	0.29	0.26	$39,100	0.06	0.06	$65,100
Medium (10-20%)	0.32	0.27	$41,700	0.07	0.06	$56,700
High (≥20%)	0.36	0.29	$55,500	0.08	0.07	$64,400
**B. UK healthcare setting**						
**By CKD stage at baseline**						
CKD stage 3B[c]	0.28	0.25	£4,300	0.07	0.06	£12,500
CKD stage 4	0.42	0.33	£10,800	0.09	0.07	£16,000
CKD stage 5, not on dialysis	0.37	0.29	£19,300	0.09	0.07	£23,900
**By 5-year risk of cardiovascular disease at baseline**						
Low (<10%)	0.33	0.29	£8,400	0.08	0.07	£17,800
Medium (10-20%)	0.36	0.29	£9,700	0.08	0.07	£15,200
High (≥20%)	0.40	0.29	£14,400	0.09	0.07	£17,800

LDL-C, low-density lipoprotein cholesterol; QALY, quality-adjusted life-year; UK, United Kingdom; US, United States

[a]Atorvastatin 20 mg daily was projected to produce only slightly smaller health benefits at similar additional cost per QALY to atorvastatin 40 mg daily (see Supplementary Tables S4 and S7 for detailed results) and could be considered as an alternative less intensive treatment option.

[b]Costs and effects discounted at 3% per annum (US) or 3.5% per annum (UK).

[c]338 (17%) of participants with CKD stage 3A (estimated glomerular filtration rate [eGFR] 60-45 mL/min/1.73 m^2).
