# Supplementary material for: Cost-effectiveness of lipid lowering with statins and ezetimibe in chronic kidney disease
Source: Kidney Int. 2019 Jul;96(1):170–9. doi: 10.1016/j.kint.2019.01.028 (PMC6595178; doi:10.1016/j.kint.2019.01.028)
Supplement: Table S10 — Sensitivity analysis of cost-effectiveness of lifetime use of atorvastatin and ezetimibe treatments in moderate-to-advanced non-dialysis chronic kidney disease (CKD) patients under different scenarios of compliance with treatment. [file mmc10.pdf]

Table S10 Sensitivity analysis of cost-effectiveness of lifetime use of atorvastatin and ezetimibe treatments in moderate-to-advanced nondialysis chronic kidney disease (CKD) patients under different scenarios of compliance with treatment

Compliance with treatment (%)	40%			60%			80%			100% (base case)		
	Life-years gained	QALYs gained	Additional cost per QALY[a]	Life-years gained	QALYs gained	Additional cost per QALY[a]	Life-years gained	QALYs gained	Additional cost per QALY[a]	Life-years gained	QALYs gained	Additional cost per QALY[a]
**A. US healthcare setting**												
**Atorvastatin 40mg daily[b] compared to no LDL-C lowering treatment**												
**By CKD stage at baseline**												
CKD stage 3B[c]	0.11	0.10	$19,600	0.16	0.14	$19,800	0.21	0.19	$20,100	0.26	0.23	$20,300
CKD stage 4	0.16	0.13	$43,900	0.23	0.19	$44,000	0.30	0.25	$44,100	0.37	0.31	$44,200
CKD stage 5, not on dialysis	0.13	0.11	$77,800	0.19	0.16	$77,900	0.25	0.21	$78,000	0.31	0.26	$78,200
**By 5-year risk of cardiovascular disease at baseline**												
Low (<10%)	0.12	0.11	$37,800	0.18	0.16	$37,900	0.23	0.21	$38,000	0.29	0.26	$38,100
Medium (10-20%)	0.13	0.12	$40,700	0.20	0.17	$40,800	0.26	0.22	$40,900	0.32	0.27	$41,000
High (≥20%)	0.15	0.12	$54,500	0.22	0.18	$54,700	0.29	0.23	$54,900	0.36	0.29	$55,000
**Ezetimibe 10mg plus atorvastatin 40mg daily compared to atorvastatin 40mg daily**												
**By CKD stage at baseline**												
CKD stage 3B[c]	0.03	0.02	$39,800	0.04	0.03	$41,000	0.05	0.04	$42,300	0.06	0.05	$43,600
CKD stage 4	0.04	0.03	$55,900	0.05	0.05	$56,700	0.07	0.06	$57,500	0.08	0.07	$58,400
CKD stage 5, not on dialysis	0.03	0.03	$89,200	0.05	0.04	$90,000	0.06	0.05	$90,700	0.07	0.06	$91,500

**By 5-year risk of cardiovascular disease at baseline**												
Low (<10%)	0.03	0.03	$61,000	0.04	0.04	$62,300	0.05	0.05	$63,600	0.06	0.06	$65,100
Medium (10-20%)	0.03	0.03	$54,200	0.05	0.04	$55,000	0.06	0.05	$55,800	0.07	0.06	$56,700
High (≥20%)	0.04	0.03	$62,400	0.05	0.04	$63,000	0.07	0.06	$63,700	0.08	0.07	$64,400
**B. UK healthcare setting**												
**Atorvastatin 40mg daily[b] compared to no LDL-C lowering treatment**												
**By CKD stage at baseline**												
CKD stage 3B[c]	0.12	0.10	£3,600	0.17	0.15	£3,700	0.23	0.20	£3,800	0.28	0.25	£3,800
CKD stage 4	0.18	0.14	£10,400	0.26	0.20	£10,400	0.34	0.27	£10,500	0.42	0.33	£10,500
CKD stage 5, not on dialysis	0.15	0.12	£18,800	0.23	0.18	£18,800	0.30	0.24	£18,900	0.37	0.29	£18,900
**By 5-year risk of cardiovascular disease at baseline**												
Low (<10%)	0.14	0.12	£7,800	0.21	0.18	£7,800	0.27	0.24	£7,900	0.33	0.29	£7,900
Medium (10-20%)	0.15	0.12	£9,300	0.22	0.18	£9,300	0.29	0.24	£9,300	0.36	0.29	£9,400
High (≥20%)	0.16	0.12	£14,000	0.24	0.18	£14,100	0.32	0.24	£14,100	0.40	0.29	£14,200
**Ezetimibe 10mg plus atorvastatin 40mg daily compared to atorvastatin 40mg daily**												
**By CKD stage at baseline**												
CKD stage 3B[c]	0.03	0.03	£11,100	0.04	0.04	£11,600	0.05	0.05	£12,000	0.07	0.06	£12,500
CKD stage 4	0.04	0.03	£15,000	0.06	0.05	£15,300	0.08	0.06	£15,600	0.09	0.07	£16,000
CKD stage 5, not on dialysis	0.04	0.03	£23,100	0.05	0.04	£23,300	0.07	0.06	£23,600	0.09	0.07	£23,900
**By 5-year risk of cardiovascular disease at baseline**												
Low (<10%)	0.03	0.03	£16,300	0.05	0.04	£16,800	0.06	0.05	£17,300	0.08	0.07	£17,800
Medium (10-20%)	0.04	0.03	£14,300	0.05	0.04	£14,600	0.07	0.06	£14,900	0.08	0.07	£15,200

High (≥20%)	0.04	0.03	£17,000	0.06	0.04	£17,300	0.08	0.06	£17,500	0.09	0.07	£17,800

LDL-C, low-density lipoprotein cholesterol; QALY, quality-adjusted life-year; UK, United Kingdom; US, United States

The analyses use country-specific non-vascular mortality rates and QoL utilities' value sets.

[a]Costs and outcomes discounted at 3% per annum (US) and at 3.5% per annum (UK).

[b]Atorvastatin 20mg daily was projected to produce only slightly smaller health benefits at similar additional cost per QALY to atorvastatin 40mg daily (see Supplementary Tables S4 and S7 for detailed results) and could be considered as an alternative less intensive treatment option.

[c]338 (17%) of participants with CKD stage 3A (estimated glomerular filtration rate [eGFR] 60-45 mL/min/1.73 m^2).
