# Supplementary material for: Cost-effectiveness of lipid lowering with statins and ezetimibe in chronic kidney disease
Source: Kidney Int. 2019 Jul;96(1):170–9. doi: 10.1016/j.kint.2019.01.028 (PMC6595178; doi:10.1016/j.kint.2019.01.028)
Supplement: Table S11 — Annual rates of nonvascular death in moderate-to-advanced chronic kidney disease (CKD) patients (United States). [file mmc11.pdf]

Table S11 Annual rates of non-vascular death in moderate-to-advanced chronic kidney disease (CKD) patients (United States).

Age, years	Men					Women				
	CKD stage 3B	CKD stage 4	CKD stage 5, not on RRT	Transplant	Dialysis	CKD stage 3B	CKD stage 4	CKD stage 5, not on RRT	Transplant	Dialysis
40-44	0.4%	0.6%	0.7%	0.8%	3.9%	0.2%	0.4%	0.4%	0.8%	3.9%
45-49	0.7%	1.0%	1.1%	2.4%	7.8%	0.4%	0.6%	0.7%	2.4%	7.8%
50-54	0.7%	1.0%	1.1%	2.4%	7.8%	0.4%	0.6%	0.7%	2.4%	7.8%
55-59	1.2%	1.6%	2.2%	2.4%	7.8%	0.7%	1.0%	1.3%	2.4%	7.8%
60-64	1.2%	1.6%	2.2%	2.4%	7.8%	0.7%	1.0%	1.3%	2.4%	7.8%
65-69	2.0%	2.8%	4.1%	5.3%	13.2%	1.3%	1.9%	2.7%	5.3%	13.2%
70-74	2.0%	2.8%	4.1%	5.3%	13.2%	1.3%	1.9%	2.7%	5.3%	13.2%
75-79	4.2%	5.9%	9.6%	9.8%	21.0%	3.0%	4.2%	6.8%	9.8%	21.0%
80-84	4.2%	5.9%	9.6%	9.8%	21.0%	3.0%	4.2%	6.8%	9.8%	21.0%
85+	9.5%	12.4%	22.6%	9.8%	21.0%	8.2%	10.7%	19.6%	9.8%	21.0%

RRT, renal replacement therapy; US, United States

Non-vascular mortality rates were derived from US-wide mortality data. For those on dialysis or on kidney transplant, mortality data were obtained directly from the 2011-2013 data from US Renal Data System[a]. For non-RRT stages, age- and gender- specific all-cause mortality rates of general population in US were adjusted using the increased hazard associated with CKD[b, c]. The non-vascular mortality rates were calculated assuming ratio of vascular to non-vascular mortality as reported in the MRC Older People study[d].

[a]U.S. Renal Data System. USRDS 2015 Annual Data Report. Volume 2: End-Stage Renal Disease in the United States. National Institutes of Health, National Institute of Diabetes and Digestive and Kidney Diseases, Bethesda(MD);2015.

[b]de Lusignan S, Chan T, Gallagher H, et al. Chronic kidney disease management in southeast England: a preliminary cross-sectional report from the QICKD–quality improvement in chronic kidney disease study. Prim Care Cardiovasc J. 2009;33(9):33-39.

[c]O'Hare AM, Bertenthal D, Covinsky KE, et al. Mortality risk stratification in chronic kidney disease: one size for all ages? Journal of the American Society of Nephrology: JASN. 2006;17(3):846-853.

[d]Chronic Kidney Disease Prognosis Consortium, Matsushita K, van der Velde M, et al. Association of estimated glomerular filtration rate and albuminuria with all-cause and cardiovascular mortality in general population cohorts: a collaborative meta-analysis. Lancet. 2010;375(9731):2073-2081
