# Supplementary material for: Cost-effectiveness of lipid lowering with statins and ezetimibe in chronic kidney disease
Source: Kidney Int. 2019 Jul;96(1):170–9. doi: 10.1016/j.kint.2019.01.028 (PMC6595178; doi:10.1016/j.kint.2019.01.028)
Supplement: Table S12 — United States (US) annual hospital care costs in moderate-to-advanced chronic kidney disease (CKD). [file mmc12.pdf]

Table S12 United States (US) annual hospital care costs in moderate-to-advanced chronic kidney disease (CKD)

	Mean cost (SE), US$	Statistical distribution in probabilistic sensitivity analysis
**Annual hospital case costs in the absence of cardiovascular complications**		
**In CKD stage 3B[a]**	5 203 (442)	Gamma
**In CKD stage 4[a]**	6 746 (3,279)	Gamma
**In CKD stage 5[a]**	6 746 (3,279)	Gamma
**On functioning kidney transplant from the current annual period**	143 889[b] (7,460[a])	Normal
**On functioning kidney transplant from an earlier annual period**	28 412[b] (1,470[a])	Normal
**On maintenance dialysis**	99 644[b] (5,196[a])	Normal
**Additional annual hospital care costs associated with death or cardiovascular complications**		
**Vascular death in the current annual period[c]**	2 047 (145)	Gamma
**Non-vascular death in the current annual period[c]**	248 (34)	Gamma
**Non-fatal MVE in the current annual period[c]**	13 495 (425)	Gamma
**Non-fatal MVE in the preceding annual period [c]**	185 (18)	Gamma
**Non-fatal MVE two or more years previously or baseline vascular disease[c]**	125 (13)	Gamma

SE, standard error; MVE, major vascular event.

Costs reported for year 2010 were updated to year 2015 using the inflation factor of 1.154 obtained from Bureau of Labor Statistics. Consumer Price Index. 2016; http://www.bls.gov/cpi/cpid1512.pdf. Cost while being on dialysis was halved for patients dying in the annual period.

[a]Erickson KF, Japa S, Owens DK, Chertow GM, Garber AM, Goldhaber-Fiebert JD. Cost-effectiveness of statins for primary cardiovascular prevention in chronic kidney disease. J Am Coll Cardiol. 2013;61(12):1250-1258.

[b]U.S. Renal Data System, USRDS 2013 Annual Data Report: Atlas of Chronic Kidney Disease and End-Stage Renal Disease in the United States. National Institutes of Health, National Institute of Diabetes and Digestive and Kidney Diseases, Bethesda(MD). 2013.

[c]Heart Protection Study Collaborative Group. Statin cost-effectiveness in the United States for people at different vascular risk levels. Circ Cardiovasc Qual Outcomes. 2009;2(2):65-72.
