# Supplementary material for: Cost-effectiveness of lipid lowering with statins and ezetimibe in chronic kidney disease
Source: Kidney Int. 2019 Jul;96(1):170–9. doi: 10.1016/j.kint.2019.01.028 (PMC6595178; doi:10.1016/j.kint.2019.01.028)
Supplement: Table S13 — Health-related quality of life in moderate-to-advanced chronic kidney disease (CKD): a linear regression model derived from Study of Heart and Renal Protection (SHARP) participant data using United States (US) EQ-5D value set. [file mmc13.pdf]

Table S13 Health-related quality of life in moderate-to-advanced chronic kidney disease (CKD): a linear regression model derived from Study of Heart and Renal Protection (SHARP) participant data using United States (US) EQ-5D value set

		Effect on quality of life, as measured by EQ-5D US utility (95% CI)
**Participant's characteristics at entry into SHARP**		
Intercept[a]		0.93 (0.92, 0.95)
Sex	Male	0.04 (0.03, 0.05)
Smoker (ref: never)	Former	-0.01 (-0.02, 0.00)
	Current	-0.03 (-0.04, -0.01)
Education level (ref: A-levels or above)	Secondary/vocational	-0.01 (-0.02, 0.00)
	Below secondary	-0.02 (-0.04, -0.01)
	Missing	-0.02 (-0.03, -0.01)
BMI, kg/m^2 (ref: ≥25, <30)	<25	0.01 (0.00, 0.02)
	≥30	-0.03 (-0.04, -0.02)
Previous failed kidney transplant	Yes	-0.05 (-0.08, -0.02)
Diabetic nephropathy	Yes	-0.04 (-0.06, -0.03)
**Participant's characteristics during SHARP**		
Age (centred at 60 years)	Per 10 years older	-0.04 (-0.04, -0.03)
Being on dialysis	Yes	-0.04 (-0.05, -0.03)
Previous (most recent) MVE (ref: no MVE within trial, no baseline vascular disease)	No MVE within trial, with baseline vascular disease	-0.05 (-0.07, -0.03)
	MVE within trial, last year	-0.13 (-0.16, -0.09)
	MVE within trial, >1 year ago	-0.07 (-0.09, -0.05)

BMI, body-mass index; CI, confidence interval; MVE, major vascular event.

[a]The intercept term corresponds to the quality of life (i.e. EQ-5D utility) of a 60-year old white female with CKD but not on dialysis, non-smoker, with education attainment of A-levels or above (or equivalent), BMI ≥25 <30 kg/m^2, without previously failed transplant, without diabetic nephropathy and without history of vascular disease.
