# Supplementary material for: Cost-effectiveness of lipid lowering with statins and ezetimibe in chronic kidney disease
Source: Kidney Int. 2019 Jul;96(1):170–9. doi: 10.1016/j.kint.2019.01.028 (PMC6595178; doi:10.1016/j.kint.2019.01.028)
Supplement: Figure S1 — Additional quality-adjusted life-years (QALYs), hospital care costs, and cost-effectiveness of lifetime use of atorvastatin and ezetimibe treatments in categories of moderate-to-advanced nondialysis chronic kidney disease (CKD) patients, by CKD stage and cardiovascular risk at baseline. [file mmc14.pdf]

Figure S1 Additional quality-adjusted life-years (QALYs), hospital care costs, and cost-effectiveness of lifetime use of atorvastatin and ezetimibe treatments in categories of moderate-to-advanced nondialysis chronic kidney disease (CKD) patients, by CKD stage and cardiovascular risk at baseline

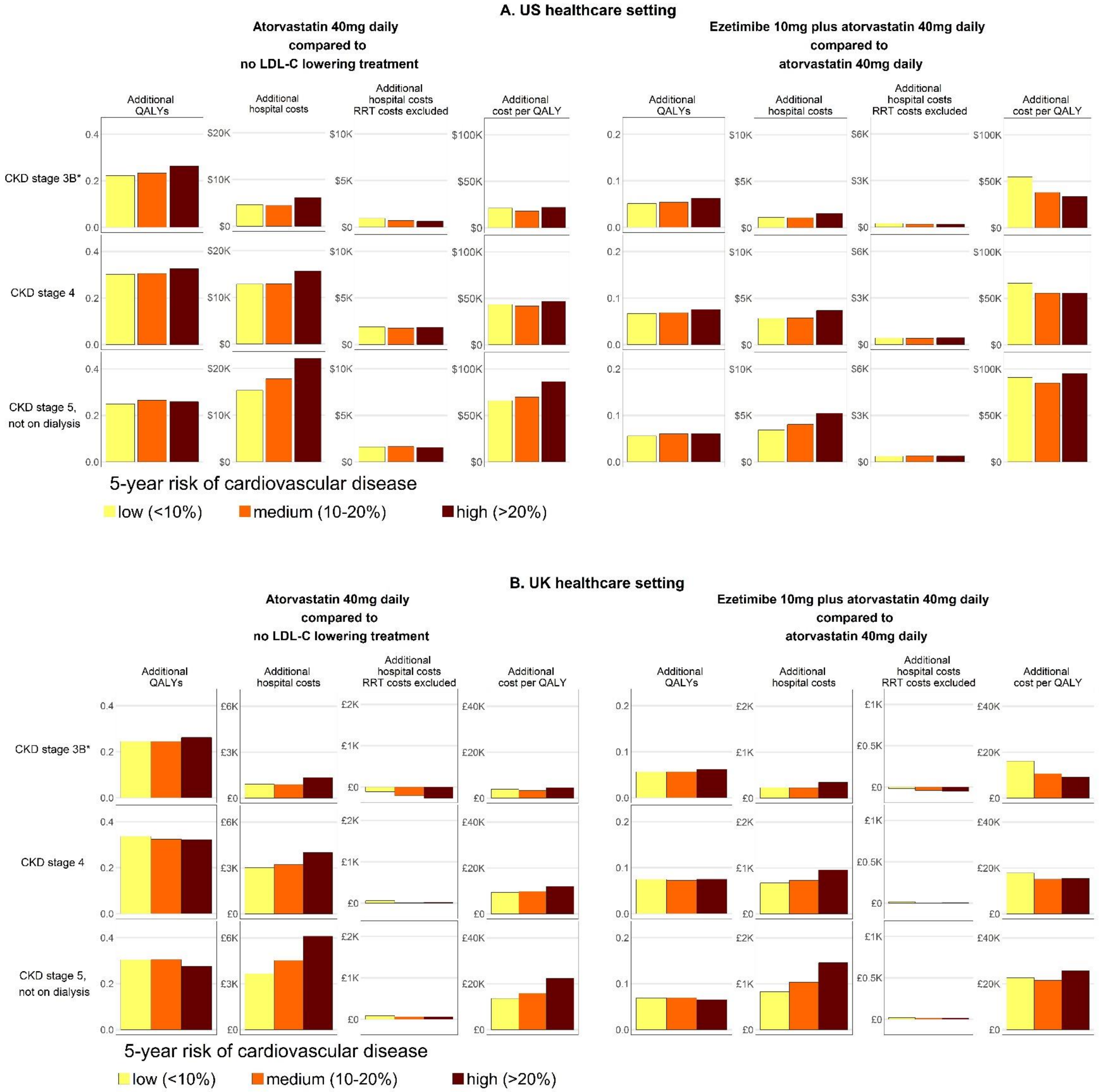

*338 (17%) of participants with CKD stage 3A (estimated glomerular filtration rate [eGFR] 60-45 mL/min/1.73 m2).
UK, United Kingdom; US, United States.
