# Supplementary material for: Cost-effectiveness of lipid lowering with statins and ezetimibe in chronic kidney disease
Source: Kidney Int. 2019 Jul;96(1):170–9. doi: 10.1016/j.kint.2019.01.028 (PMC6595178; doi:10.1016/j.kint.2019.01.028)
Supplement: Figure S2 — Schematic of the Study of Heart and Renal Protection (SHARP) CKD-CVD lifetimes health outcomes model. [file mmc15.pdf]

Figure S2 Schematic of the Study of Heart and Renal Protection (SHARP) CKD-CVD lifetimes health outcomes model

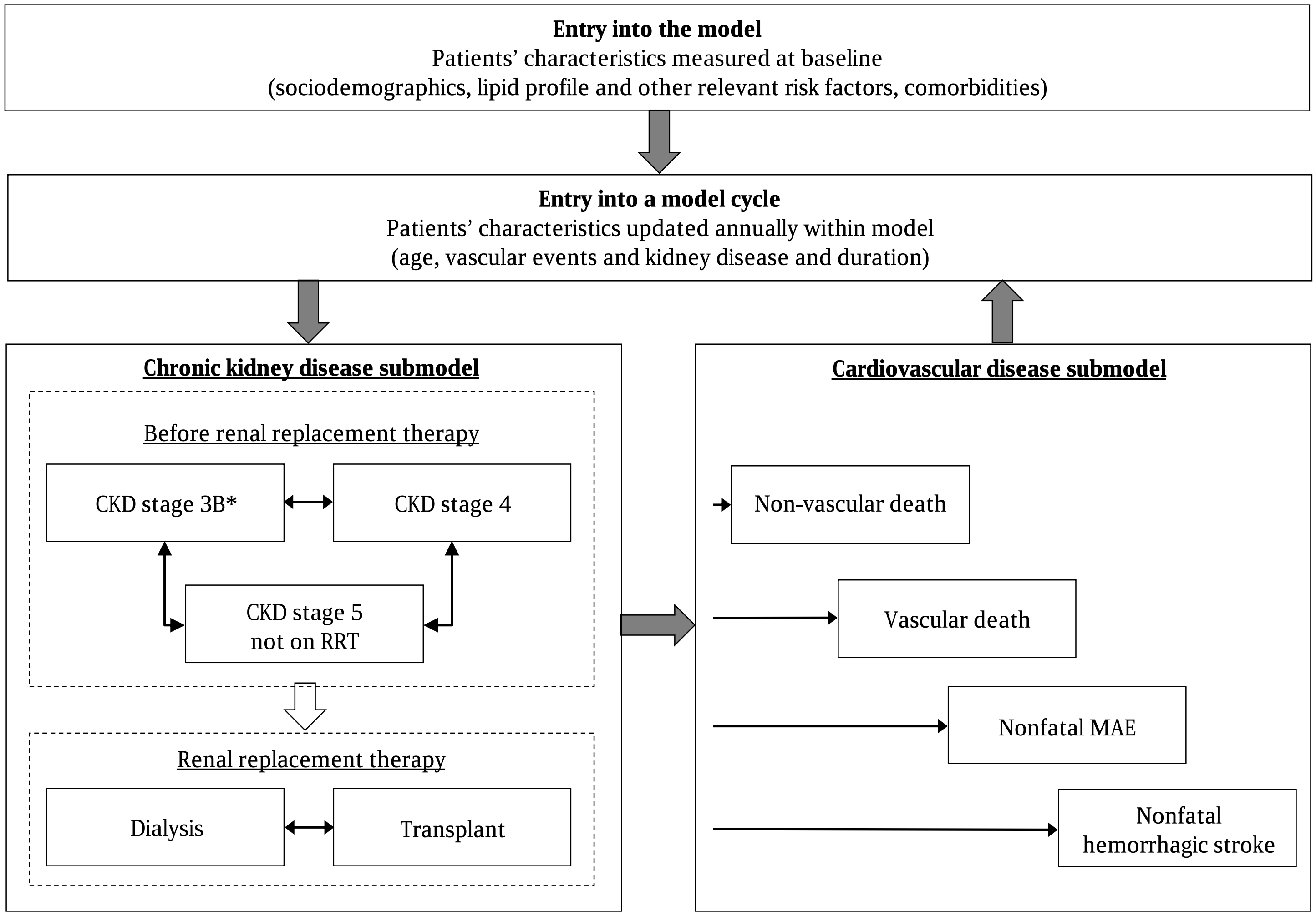

*338 (17%) of participants with CKD stage 3A (estimated glomerular filtration rate [eGFR] 60-45 mL/min/1.73 m^2).
CKD, chronic kidney disease; MAE, major atherosclerotic event; RRT, renal replacement therapy.
