# Supplementary material for: Cost-effectiveness of lipid lowering with statins and ezetimibe in chronic kidney disease
Source: Kidney Int. 2019 Jul;96(1):170–9. doi: 10.1016/j.kint.2019.01.028 (PMC6595178; doi:10.1016/j.kint.2019.01.028)
Supplement: Figure S3 — Schematic of the information sources of the Study of Heart and Renal Protection (SHARP) CKD-CVD lifetime health outcomes model. [file mmc16.pdf]

Figure S3 Schematic of the information sources of the Study of Heart and Renal Protection (SHARP) CKD-CVD lifetime health outcomes model

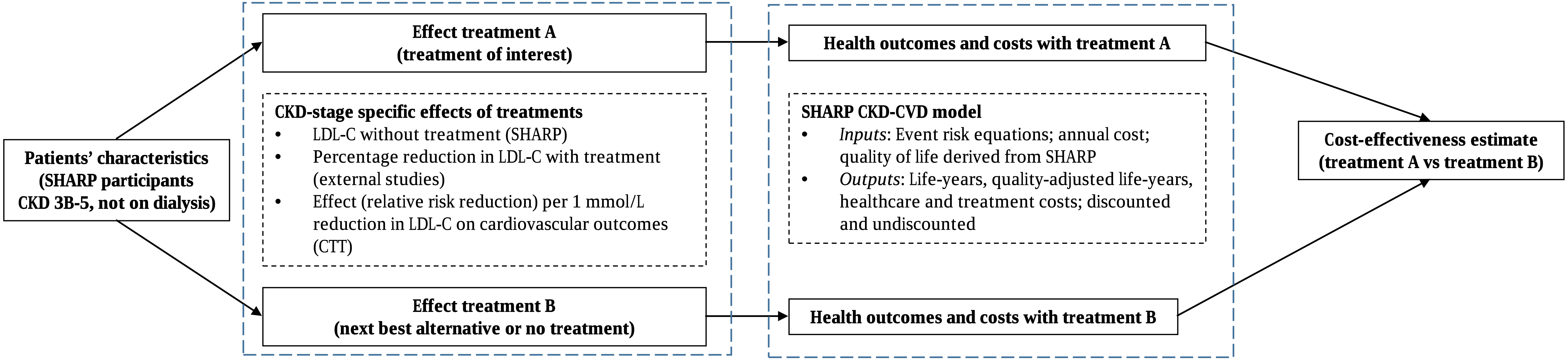

CKD, chronic kidney disease; CTT, Cholesterol Treatment Trialists, LDL-C, low-density lipoprotein cholesterol.
